# Kinetic analysis of ATP hydrolysis by complex V in four murine tissues: Towards an assay suitable for clinical diagnosis

**DOI:** 10.1371/journal.pone.0221886

**Published:** 2019-08-28

**Authors:** Francis Haraux, Anne Lombès

**Affiliations:** 1 Institute for Integrative Biology of the Cell (I2BC), CEA, Gif-sur-Yvette, France; 2 UMR 9198, CNRS, Gif-sur-Yvette, France; 3 Université Paris-Sud, Université Paris-Saclay, Gif-sur-Yvette, France; 4 Institut Cochin, Unité U1016, INSERM, Paris, France; 5 UMR 8104, CNRS, Paris, France; 6 Université Paris 5, Paris, France; Charite Universitatsmedizin Berlin, GERMANY

## Abstract

**Background:**

ATP synthase, the mitochondrial complex V, plays a major role in bioenergetics and its defects lead to severe diseases. Lack of a consensual protocol for the assay of complex V activity probably explains the under-representation of complex V defect among mitochondrial diseases. The aim of this work was to elaborate a fast, simple and reliable method to check the maximal complex V capacity in samples relevant to clinical diagnosis.

**Methods:**

Using homogenates from four different murine organs, we tested the use of dodecylmaltoside, stability of the activity, linearity with protein amount, sensitivity to oligomycin and to exogenous inhibitory factor 1 (IF1), influence of freezing, and impact of mitochondrial purification.

**Results:**

We obtained organ-dependent, reproducible and stable complex V specific activities, similar with fresh and frozen organs. Similar inhibition by oligomycin and exogenous IF1 demonstrated tight coupling between F_1_ and F_0_ domains. The Michaelis constant for MgATP had close values for all organs, in the 150–220 μM range. Complex V catalytic turnover rate, as measured in preparations solubilized in detergent using immunotitration and activity measurements, was more than three times higher in extracts from brain or muscle than in extracts from heart or liver. This tissue specificity suggested post-translational modifications. Concomitant measurement of respiratory activities showed only slightly different complex II/complex V ratio in the four organs. In contrast, complex I/complex V ratio differed in brain as compared to the three other organs because of a high complex I activity in brain. Mitochondria purification preserved these ratios, except for brain where selective degradation of complex I occurred. Therefore, mitochondrial purification could introduce a biased enzymatic evaluation.

**Conclusion:**

Altogether, this work demonstrates that a reliable assay of complex V activity is perfectly possible with very small samples from frozen biopsies, which was confirmed using control and deficient human muscles.

## Introduction

Mitochondrion is the power station of the cell. It converts the redox energy contained in food metabolites into the phosphate bond energy of ATP, the universal fuel of living organisms. This process, called oxidative phosphorylation or OXPHOS, occurs at the level of the inner mitochondrial membrane. Four enzymatic complexes (I, II, III and IV), associated with the mobile carriers ubiquinone and cytochrome c, transfer electrons from reducing substrates to oxygen. Complexes I, III and IV couple redox reactions to proton flow from the matrix to the intermembrane space, thus giving rise to the protonmotive force (pmf). F_0_F_1_ ATP synthase (complex V) uses the exergonic proton backflow for ATP synthesis from ADP and inorganic phosphate, which occurs in the matrix. Specific carriers exchange ATP against ADP and transport phosphate across the inner mitochondrial membrane. In the absence of pmf, the reverse reaction of ATP hydrolysis should occur but is down regulated by the Inhibitory Factor 1 (IF1), an endogenous peptide present in the matrix [1, 2]. This molecule, which contains 81 aminoacids in human, binds to the catalytic domain of complex V when the pmf collapses. It is released when the pmf is restored, allowing ATP synthesis.

Complex V is composed of 17 different subunits, organized in two domains: F_1,_ a matrix, ATP-synthetizing domain, and F_0_, a membranous, proton-channel domain [3]. Two stalks link these two domains, coupling proton transfer to ATP synthesis or hydrolysis. In mammals, only two structural subunits of complex V (a and A6L, both located in the F_0_ domain) are encoded by genes of the mitochondrial DNA (mtDNA) while the rest of the subunits, as well as all assembly factors, are encoded by nuclear DNA genes.

Mitochondrial diseases are defined as the diseases due to defective OXPHOS machinery. They are among the most frequent inherited metabolic diseases, with an incidence evaluated around 1/6000 [4]. Identified genetic alterations causing human ATP synthase defects affected nine different genes: the two mitochondrial DNA genes encoding F_0_ structural subunits (*MT-ATP6* and *MT-ATP8* [5]) and seven nuclear genes encoding either assembly factors (*ATP12*, *TMEM70* [6, 7]), structural subunits of the F_1_ domain (*ATP5F1E*, *ATP5F1A*, *ATP5F1D* [8–11]) or *OXA1L*, a mitochondrial insertase, whose alteration causes a combined OXPHOS defect including complex V [12].

Complex V defects appear as under-represented among mitochondrial diseases. One important difficulty of their diagnosis resides in the lack of a consensual protocol for the assay of complex V activity. ATP production is the physiological function of complex V. Direct quantification of its rate requires either fresh biopsy or cultured cells as well as permeabilization to give access to saturating amounts of substrates and ADP. A defective rate cannot differentiate defect in complex V from defect in substrate oxidation by the respiratory chain. In addition, that approach cannot measure the maximal turn-over rate of ATP synthesis, which requires preparing tightly coupled submitochondrial particules, applying a very high protonmotive force, and showing that it is saturating (i.e. slightly lowering the pmf does not changes the rate of ATP synthesis) [13]. Spectrophotometric assays of the reverse ATP hydrolysis reaction, coupled to NADH oxidation by an enzymatic ATP-regenerating system, have the great advantage to be possible on frozen samples and to measure the activity at its maximal rate. In the literature, these different approaches have been applied but rarely compared [8, 10–12]. In few instances, they have brought divergent results [14, 15].

To try to improve the diagnosis of human ATP synthase defects, we addressed the protocol of the spectrophotometric assay of ATP hydrolysis. We analyzed four different murine tissues: brain, liver, muscle and heart that are relevant to clinical investigation in human (muscle, liver) or are known targets in diseases due to ATP synthase defect (brain, heart). In these tissues, we studied the use of dodecylmaltoside (DDM), the impact of freezing, the presence of ectopic non-mitochondrial ATP synthase, and the kinetics of the enzyme that could underlie the tissue specific expression previously associated with generalized ATP synthase defect. We could set up a reliable assay of complex V activity, compatible with very small samples from frozen biopsies. We confirmed its applicability in human muscle biopsies and analyzed two samples with known deleterious *MT-ATP6* mutations

## Material and methods

### Samples

All patients and control subjects gave their written informed consent for sampling and analyses according to our Institutional ethics board.

All animal procedures were carried out according to French legal regulations and were approved by the ethics committee « Comité d’éthique en matière d’expérimentation animale de l’université Paris 5-Descartes » (n°CEEA34.MCAG.101.12). Wild type males with the C57BL6/J background, around one year of age, were sacrificed by decapitation. Dissection was immediate, obtaining samples from heart, quadriceps muscle, liver and brain cortex. These samples were either stored at -80°C before use or kept at 0–4°C for less than 15 minutes before homogenization.

For homogenates preparation, 20–60 mg of tissue were put in a glass-glass Potter tissue grinder containing 300–600 μl of homogenization buffer containing 225 mM mannitol, 75 mM saccharose, 10 mM Tris HCl, 0.1 mM ethylenediaminetetraacetic acid **(**EDTA), pH 7.4, supplemented with cOmplete ^TM^ antiprotease cocktail (1 tablet for 50 ml). The sample was homogenized on ice by manual strokes, and then centrifuged at 1000 *g* for 10 minutes at 4°C. Part of the supernatant, complemented with 5 mg/ml fatty acid—free bovine serum albumin (BSA), was stored on ice before kinetic experiments. The ATPase activity of this preparation, stored at 4°C, was stable for at least one day, sometimes for 2–3 days. Measurement of the protein concentration in the rest of the supernatant used the Pierce BCA Protein assay kit (Thermo Fisher Scientific, USA)

### Measurement of ATP hydrolysis

A sketch of the assay is provided in S1 Fig 1.5 to 10 μl sample was put into a 1 ml spectrophotometric cuvette, at 37°C, containing 250 mM mannitol, 50 mM Tris, 10 mM KCl, 5 mM MgCl_2_,1 mM ethylene glycol-bis(β-aminoethyl ether)-*N*,*N*,*N*′,*N*′-tetraacetic acid (EGTA), 1 mg/ml BSA, pH 8.25, supplemented with 0.4 mM NADH, 1 μM antimycin, 1 μM carbonyl cyanide p-trifluoromethoxyphenylhydrazone (FCCP), 1 mM phosphoenolpyruvate **(**PEP), 10 units lactodehydrogenase (LDH), and 25 units pyruvate kinase (PK). When indicated, 0.01% w/w dodecylmaltoside (DDM) and 3 μM P1,P5-Di(adenosine-5')pentaphosphate **(**Ap5A) were added. All reagents were in highly concentrated stock solutions allowing additions as small volumes, such as total added volume never exceeded 50 μl. Absorbance was continuously measured at 340 nm with a 1 cm optical pathway, either under stirring using a Suprasil ^TM^ 109004F-10-40 cuvette (Hellma, Germany) or without stirring using a regular plastic cuvette. In the absence of stirring, the cuvette was closed with thumb using a piece of Parafilm ^TM^ and rapidly shaken (at least four reversals) after each addition. Interruption of the measurement, due to addition and mixing, never exceeded 5–10 seconds. Stirred and unstirred conditions gave the same results. After 3–5 minutes of incubation in the spectrophotometer, the reaction was triggered by adding 1 mM MgATP or less when indicated. Kinetics of ATP hydrolysis was analyzed as explained in the Results section using ε_M_ = 6220 M^-1^ cm^-1^. When indicated, 3 μM IF1 or 6 μM oligomycin were added. For K_m_ determinations, MgATP concentration was corrected for traces of ATP and ADP present in biological samples as explained in S2 Fig.

### Measurement of complex I activity

The protocol was adapted from [16]. 1 ml spectrophotometric cuvette containing 50 mM K-phosphate, 2 mg/ml BSA, pH 7.5, supplemented with 100 μM NADH, 0.01% w/w DDM, and 100 μM decyl-ubiquinone, was put into the spectrophotometer (λ = 340 nm, optical pathway 1 cm), with temperature controlled at 37°C. Some absorbance decrease generally occurred after mixing the reagents. After some minutes, the signal became stable and addition of some μl of sample triggered the reaction. The activity decayed with time to a level close to zero. We discarded the first 10 seconds of the kinetics, perturbed by the sample addition. Fitting with a monoexponential equation using the solver of Microsoft Excel^TM^ software gave a first estimate of the initial rate and the value of the final rate [17]. The initial rate was then refined by a second fit restricted to the first three minutes following the sample addition, keeping fixed the previously determined value of the final rate. We subtracted from the initial rate the value obtained in control experiments carried out in the absence of decyl-ubiquinone and in the presence of 12.5 μM rotenone. This correction never exceeded 10% of the total rate value. The rate of NADH oxidation was calculated using ε_M_ = 6220 M^-1^ cm^-1^.

### Measurement of complex II activity

The protocol was adapted from [18]. 0.01% w/w DDM, 100 μM decylubiquinone, 50 μM 2,6-dichlorophenolindophenol (DCPIP), 1 mM potassium cyanide (KCN) and 100 μM ATP were added to the assay medium containing 25 mM K phosphate, 25 mM K succinate, 2 mg/ml BSA, pH 7.5 in a 1 mL spectrophotometer cuvette at 37°C (λ = 600 nm, optical pathway 1 cm). After baseline stabilization, addition of some μl of sample triggered the reaction. The activity was stable for several minutes. Addition of 10 mM malonate inhibited the reaction. Complex II activity was calculated as the rate of DCPIP reduction using ε_M_ = 19100 M^-1^ cm^-1^ and subtracting the small drift after malonate addition.

### Blue-Native PAGE

Blue-Native PAGE was performed as described [19]. Briefly, homogenates were centrifuged at 11000 *g* during 10 minutes at 0–4°C to obtain the crude mitochondrial pellet, which was resuspended in 100 μL in the homogenization buffer for the measurement of its protein content using the Pierce BCA Protein assay kit (Thermo Fisher Scientific, USA). After further centrifugation of aliquots of mitochondrial preparation at 11000 *g* during 10 minutes at 0–4°C, the resulting pellets were gently homogenized at 5 mg/mL in 50 mM NaCl, 50 mM imidazole/KCl, 2 mM 6-aminohexanoic acid, 1 mM EDTA, at pH 7.0 and 20% DDM solution was added up to a DDM/protein ratio of 6. After 15 minutes incubation on ice, centrifugation at 20000 *g* during 20 minutes at 0–4°C allowed obtaining the solubilized proteins in the supernatant, whose protein content was measured on an aliquot diluted ¼ in PBS using the Pierce BCA Protein assay kit (Thermo Fisher Scientific, USA). 5% Serva blue G solution (Serva, Germany) was added up to a DDM/Serva blue ratio of 8 (w/w) together with 5% glycerol. The samples were kept at -80°C until electrophoresis.

Electrophoresis was performed using XCell SureLock mini-cell electrophoresis system, NativePAGE 3–12% Bis-Tris protein gels, and native PAGE running buffers (Thermo Fisher Scientific, Germany). After running at 80 volts during 30 minutes then at 100 volts during 90 minutes, the Dark Blue Cathode buffer was replaced by Light Blue Cathode buffer and the running proceeded for a further 60 minutes at 100 volts followed by 120 minutes at 150 volts. Transfer onto an Immobilon-P membrane with 0.45 μm pore size (Thermo Fisher Scientific) was performed in 192 mM glycine, 25 mM Tris, 0.2% sodium dodecylsulfate **(**SDS), 20% ethanol at 100 volts during 90 minutes. Membranes were washed during 5 minutes in 100% isopropanol, fixed in 10% acetic acid + 25% isopropanol during 10 minutes, rinsed in PBS + 0.1% Tween 20 during 5 minutes, and blocked in PBS + 0.1% Tween 20 + 5% skimmed milk during 90 minutes. Incubation was performed overnight at 4°C with polyclonal antibodies against the F_1_ domain of bovine complex V (anti-F_1_, produced in the group of Professor Joël Lunardi, Grenoble) diluted 1/5000 in PBS + 0.1% Tween 20+2% skimmed milk. After four washes of the membranes in PBS + 0.1% Tween 20, primary antibodies were visualized using peroxidase-conjugated secondary antibodies (Sigma-Aldrich, France) diluted 1/5000 in PBS + 0.1% Tween 20 + 2% skimmed milk and Pierce™ ECL Western Blotting Substrate (Life Technologies, USA). Quantification of the luminescent signals used volumes obtained after basal adjustment with rolling ball approach by Fusion FX (Vilber Company, Germany).

### Reagents and ATPase inhibitor

All reagents were of analytic grade. The stock solutions of MgATP were prepared as follows. ATP (Sigma-Aldrich) was first prepared at the highest possible concentration, by alternatively adding ATP powder and concentrated KOH to H_2_O. The viscous solution so obtained was diluted 10000 fold to determine its nucleotide concentration by UV spectroscopy (ε_M_ = 14500 M^-1^ cm^-1^ at 258 nm). The concentrated solution was then mixed with water and 1 M MgCl_2_ in order to obtain 0.4 M MgCl_2_ and 0.4 M ATP. ATP concentration was verified by the NADP^+^ reduction using 1 mM glucose, 1 mM NADP^+^, 10 units/ml hexokinase and 10 units/ml glucose-6-dehydrogenase. The absorbance at 340 nm (ε_M_ = 6220 M^-1^ cm^-1^) gave the concentration of reduced NADP^+^, equal to the concentration of ATP injected into the cuvette. As a rule, ATP concentration, evaluated by that assay, was 98–100% of the concentration evaluated by absorbance at 258 nm.

Yeast IF1 was overexpressed in *E*. *coli* and purified as previously described [17]. We also used synthetic yeast IF1 from PolyPeptide (Strasbourg, France), with the native sequence as well as a C-terminal truncated form (S3 Fig). The three peptides inhibited F_0_F_1_-ATPase activity to the same extent.

### Statistical analyses

All comparisons used non parametric Mann and Whitney test.

## Results

### A specific assay of complex V activity in murine muscle

**Fig 1:**
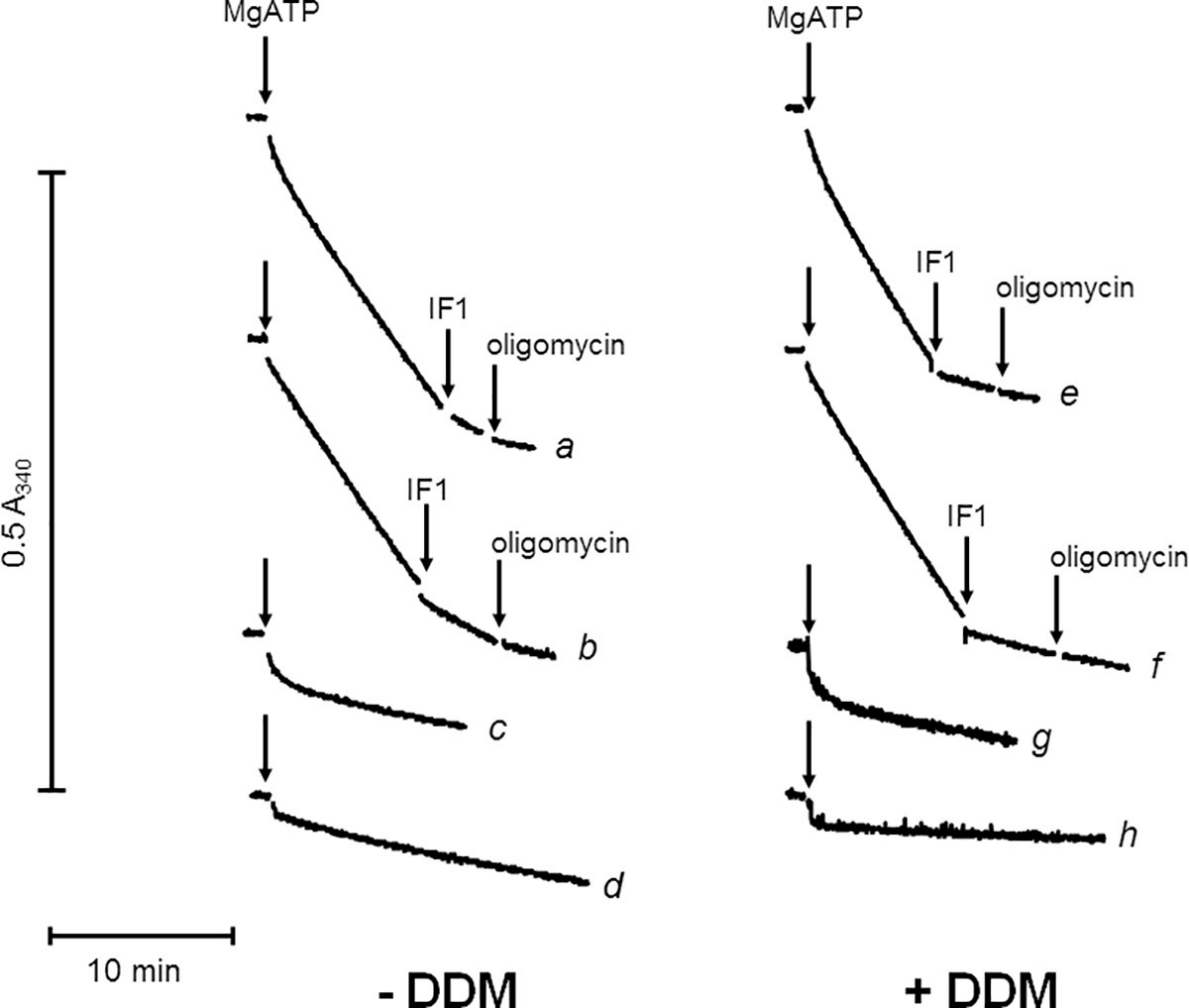
Time-course of ATP hydrolysis by muscle homogenate with and without DDM. Conditions as described under Materials and Methods; additional reagents initially present were 3 μM Ap5A (b, d, f, h), IF1 and oligomycin (c, d, g, h); 32 μg protein added in the absence of DDM (left panel, traces a-d) or in the presence of 0.01% DDM (right panel, traces e-h); vertical arrows = additions of 1 mM MgATP or 3 μM yeast IF1 or 6 μM oligomycin.

Muscle being the most frequent tissue analyzed in clinical diagnosis of mitochondrial diseases, we first addressed the assay of complex V activity in that tissue. After an instantaneous drop of absorbance due to some contaminating ADP, ATP hydrolysis by an homogenate from a frozen-thawed fragment of murine muscle followed a biphasic kinetics with a short initial rapid phase then a longer less active phase (Fig 1 curve a). We hypothesized that the initial phase was due to an adenylate kinase reaction fed by ATP and some traces of AMP, ending when AMP was exhausted. This was demonstrated by the total inhibition of this initial phase by Ap5A (curve b). Inhibition of the following steady state by the addition of yeast IF1 at a high concentration (3 μM), and even more by a subsequent oligomycin addition, demonstrated that it was due to complex V. Initial presence of IF1 and oligomycin preserved the initial phase but inhibited the steady state activity, confirming that it was complex V activity (curve c). Initial presence of Ap5A in addition to IF1 and oligomycin inhibited both phases (curve d). Even under uncoupled conditions, transport of complex V substrates (ATP, ADP and phosphate) and the endogenous inhibitor peptide IF1 may down regulate ATP hydrolysis by complex V. To test the impact of these factors, we added 0.01% DDM to the assay medium (Fig 1 right panel). DDM increased the steady state activity by about 15%, probably by the release of above-mentioned down-regulations. DDM also increased the sensitivity of complex V activity to yeast IF1, which now inhibited the activity to an extent that was not significantly increased by subsequent oligomycin addition (compare curves b and f). Disruption of some intact mitochondria in which the catalytic part of complex V was initially not accessible to externally added IF1 likely explained that observation. DDM did not affect the initial phase attributed to adenylate kinase (curves g and h).

### Complex V activity in different murine tissues

In all tissues, complex V activity was measured in the presence of DDM and Ap5A unless otherwise stated (Table 1). Homogenates prepared from frozen-thawed fragments of murine liver, and heart hydrolyzed ATP in the same way as preparations from muscle. In all cases the reaction proceeded at a constant rate and was highly sensitive to IF1 + oligomycin addition (see examples in S4 Fig). In all tissues, oligomycin alone inhibited the activity to the same extent as IF1 + oligomycin (see examples in S5 Fig), showing that DDM fully preserved the functional coupling between domains F_1_ and F_0_. Brain homogenates sometimes presented with an initial contaminating activity. In these cases, ATP hydrolysis did not proceed at the constant rate shown in Fig 1 and S4 Fig but the activity decayed to reach a steady state value after 10–20 minutes. This behavior was preparation-dependent and occurred in roughly 50% of the cases. It occurred in the presence of Ap5A and was unaffected by IF1 and oligomycin added before MgATP (see example in S6 Fig), showing that it was not related to adenylate kinase or complex V. Dilution of the assay preparation in the spectrophotometric cuvette 20 min before MgATP addition minimized the contaminating activity. In any case, the steady state ATPase activity and its sensitivity to complex V inhibitors, measured after this initial burst of activity, did not statistically differ from those measured on other brain homogenates that did not present with that contaminating activity. The complex V activity was proportional to the protein concentration (S7 Fig), indicating that endogenous IF1 inhibitory effect, expected to depend on sample concentration, was negligible due to its high dilution in the cuvette. In addition, the proportionality between activity and protein concentration at the constant DDM concentration of 0.01% also indicated that the inhibitory effect of DDM was negligible when using sufficient protein amount per assay. That amount varied with the tissue. We estimated that using at least 20 micrograms per assay for brain, 30 for heart and muscle and 40 for liver avoided entering the range of DDM/protein where DDM could become inhibitory.

**Table 1:** Mean specific complex V activity in homogenates from different organs in the presence of 0.01% DDM.

organ	complex V activity		% of crude activity		# preparations		# measurements	
	frozen	Fresh	Frozen	fresh	frozen	fresh	frozen	fresh
brain	104 ± 30	120 ± 47	70 ± 8	63 ± 11	14	4	51	15
liver	367 ± 89	271 ± 152	91 ± 3	80 ± 6	5	4	14	11
muscle	326 ± 128	468 ± 123	88 ± 5	80 ± 9	10	4	31	8
heart	1669 ± 599	1604 ± 569	96 ± 1	94 ± 3	6	5	24	11

Conditions as in Fig 1; crude activity = activity in the presence of Ap5A; complex V activity = crude activity minus residual activity after addition of IF1 and oligomycin; % of crude activity = ratio complex V activity to crude activity; complex V activity is shown as mean and standard deviation of values expressed as nmol ATP min^-1^ mg protein^-1^

# = number of preparations or measurements.

We then addressed the reproducibility of the assay in the different tissues and compared its results in fresh *versus* frozen-thawed tissue fragments (Table 1). Several experiments performed with different preparations from frozen-thawed and from fresh tissues showed that heart had the highest activity, muscle and liver intermediate activities, and brain had low activity. These values were similar in fresh or frozen/thawed tissues. Indeed, the ratio of specific activity between preparations from frozen-thawed (Table 1 2^nd^ column) and fresh (3^rd^ column) materials was about 0.85 for brain, 1.35 for liver, 0.70 for muscle and 1.04 for heart. We thus concluded that quantitative estimation of complex V activity was possible in frozen as well as fresh tissues.

### Evaluation of complex V activity in intact mitochondria

**Fig 2:**
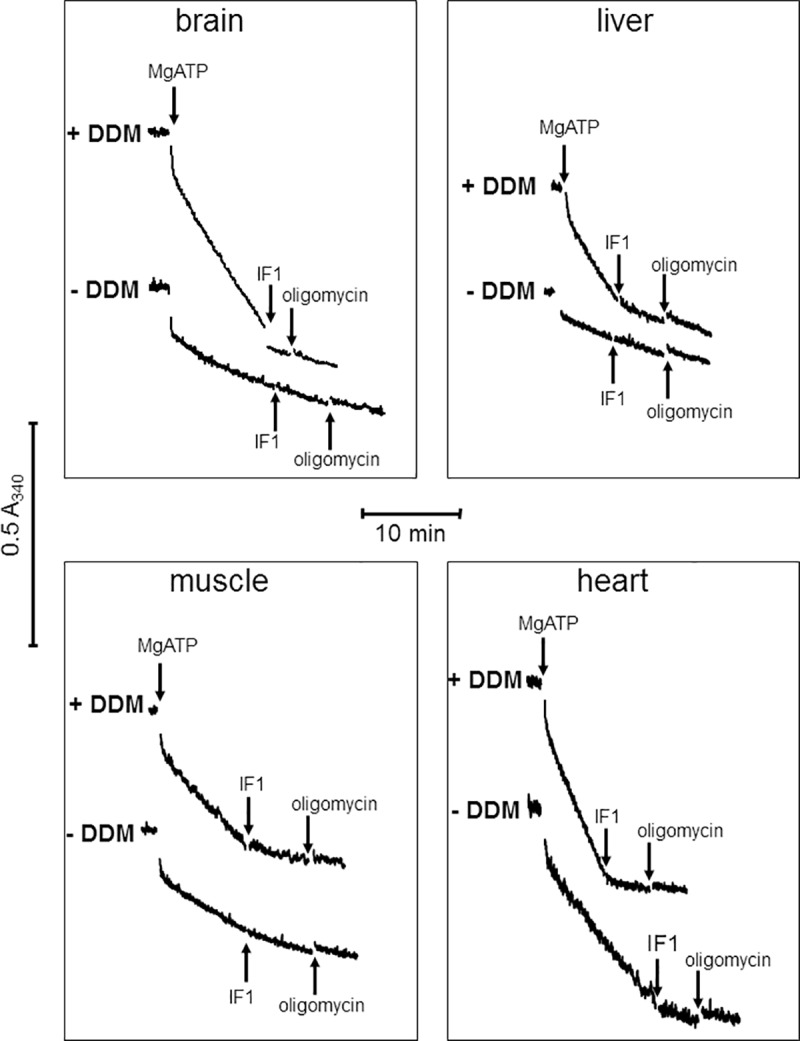
Time-course of ATP hydrolysis by homogenates from fresh organs in presence and absence of DDM. Conditions as described under Materials and Methods; vertical arrows = additions (1 mM MgATP, 3 μM yeast IF1, 6 μM oligomycin); protein content was 20.5 μg for brain, 7.2 μg for liver, 9.9 μg for muscle, and 3.1 μg for heart; complex V activity specifically sensitive to IF1 = steady state rate of ATP hydrolysis minus rate after IF1 addition; total complex V activity = steady state rate of ATP hydrolysis minus final rate after IF1 + oligomycin addition.

We used DDM because it disrupted mitochondrial membranes making complex V accessible to externally added substrates and yeast IF1. Indeed, preparations from fresh tissues, which contain an important proportion of intact mitochondria, presented with a much lower complex V activity in the absence of DDM than in its presence. Furthermore, additional inhibition brought by oligomycin after IF1 showed that added IF1 incompletely inhibited complex V activity in fresh tissue preparations (Fig 2). Heart, whose crude activity is significant in the absence of DDM, well exemplified the effect of DDM.

We used DDM effect to evaluate the fraction of mitochondria damaged during the sample preparation (Table 2 “fraction mitochondrial damage”). Normalizing the IF1-sensitive activity in the absence of DDM to that obtained in its presence estimated the fraction of complex V initially accessible to IF1 and therefore located in initially damaged mitochondria. That ratio would equal zero if all the mitochondria were intact and one if they were all damaged. In the examples depicted in Fig 2, the fraction of initially damaged mitochondria was zero for liver, 0.04 for brain, 0.27 for muscle and 0.35 for heart. The fraction of damaged mitochondria for a given tissue varied between preparations. However, preparations from muscle and heart always had more damaged mitochondria than brain and liver, most probably because of the relatively harsh homogenization required for these two tissues.

**Table 2:** Ratios between ATPase activities of complex V measured in absence and presence of DDM in homogenates of different fresh tissues.

organ	preparation	- / + DDM ratio	
		Fraction mitochondrial damage	Weighted complexV activity
brain	I	0.04	0.14
	II	0.14	0.13
liver	I	0	0.16
	II	0.16	0.34
muscle	I	0.29	0.38
	II	0.64	0.69
heart	I	0.35	0.38
	II	0.47	0.52
	III	0.59	0.60

Conditions as in Fig 2; the activity values in the presence of DDM used to calculate the ratio were the average of two determinations; complex V activity and IF1-sensitive complex V activity calculated as in Fig 2; Fraction mitochondrial damage = ratio between IF1-sensitive activity in the absence of DDM and that obtained in its presence; Weighted complex V activity = ratio between IF1+oligomycin-sensitive activity in the absence of DDM and that obtained in its presence.

In damaged mitochondria, saturating levels of exogenously added substrates allow complex V activity to proceed at maximal rate whereas, in intact mitochondria, the levels of endogenous metabolites regulate complex V activity. Complex V activity in intact mitochondria is sensitive to oligomycin but unaffected by exogenous IF1. In the absence of DDM, the oligomycin+IF1-sensitive activity therefore measures the global complex V activity, adding the maximal activity from damaged mitochondria and the regulated one from intact mitochondria. Adding DDM equaled damaging the whole mitochondrial population and allowing complex V maximal activity in all mitochondria. Therefore, normalizing the IF1+oligomycin-sensitive activity in the absence of DDM to that obtained in its presence gave a ratio estimating the relative weight in complex V activity of intact and damaged mitochondria (“Weighted complex V activity” in Table 2). If all mitochondria were damaged, the ratio would equal one. If all mitochondria were intact, it would equal the ratio between complex V activity in intact mitochondria and the maximal complex V activity obtained in damaged ones.

**Fig 3:**
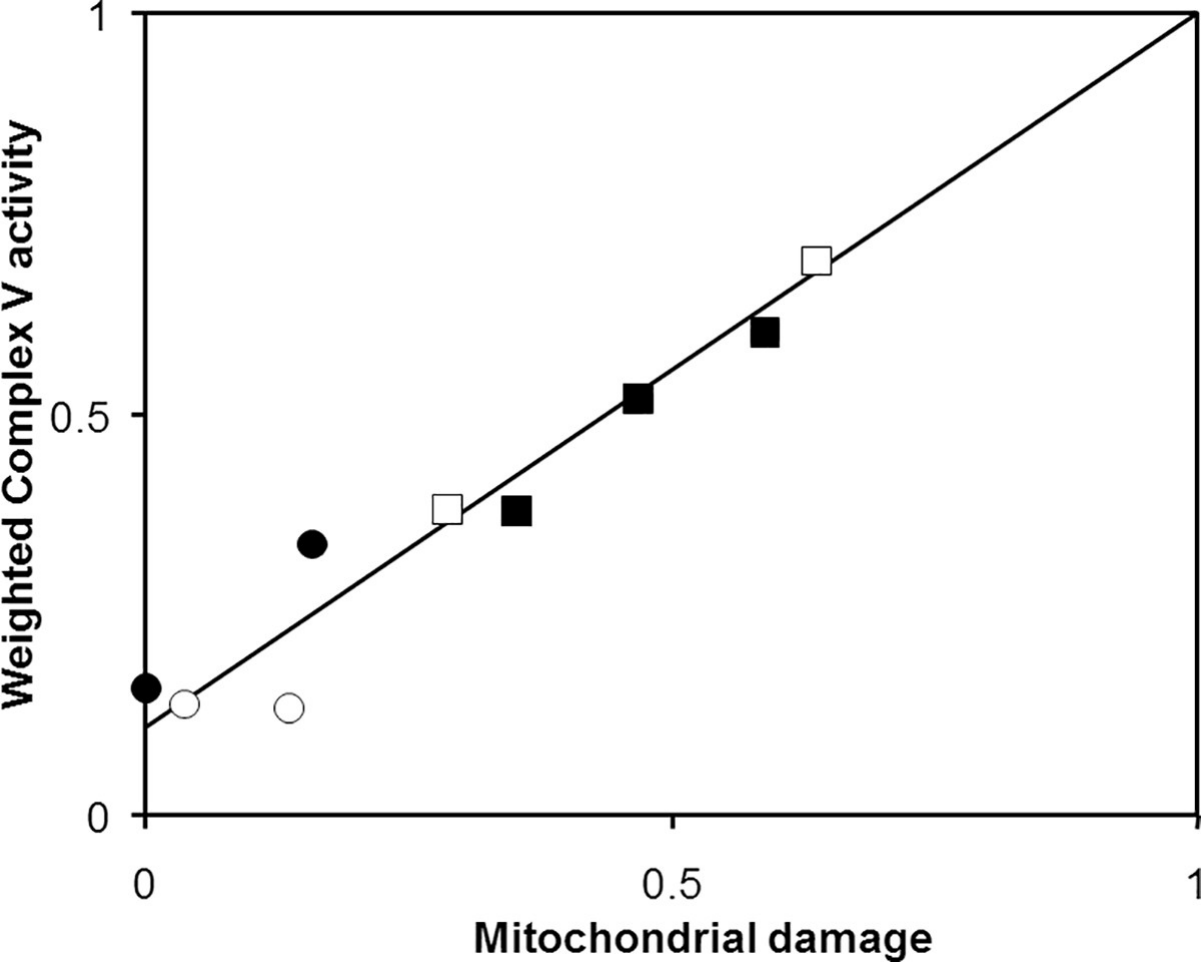
“Weighted complex V activity” as a function of “mitochondrial damage”. Data from Table 2. (○) brain; (●) liver; (□) muscle; (■) heart.; “weighted complex V activity” is given by the fraction of complex V activity measured in the absence of DDM; “mitochondrial damage” is given by the fraction of ATPase activity sensitive to external IF1 addition in the absence of DDM. Data were fitted by the linear relationship V = V_i_ + α (1-V_i_), where V is the Weighted complex V activity, V_i_ the fraction mitochondrial damage, and α the ratio between complex V activity in intact mitochondria and that in damaged ones. Here α = 0.11, which means that the complex V activity in intact mitochondria is only 11% of the activity in damaged or DDM-treated mitochondria.

Plotting the “Weighted complex V activity” against the “fraction mitochondrial damage” showed the linear relationship between these two parameters (Fig 3). The intercept represents the complex V activity in ideal preparations containing only intact mitochondria. It is the ratio (noted α) between complex V activity in intact mitochondria and that in damaged ones. The slope is 1-α. Interestingly, merging data from the four different tissues gave a common plot suggesting that the activity of complex V in intact mitochondria would be only 11% of its maximal activity.

### Catalytic properties of complex V in murine tissues

Specific activities allowed global comparison between tissues or organs but did not provide information about possible tissue-specificity of complex V function. We explored that question by studying the catalytic properties of complex V in murine brain, liver, muscle and heart. First, we determined the Michaelis constant (K_m_) for MgATP in homogenates from these different organs (S8 Fig). The mean K_m_ values obtained by merging data from different preparations were: 148 ± 12 μM (brain); 220 ± 22 μM (liver); 217 ± 19 μM (muscle); 146 ± 11 μM (heart). These values did not significantly differ from each other. Therefore, if catalytic properties of complex V differed according to its tissue localization, it did not result in affinity change for MgATP.

**Fig 4:**
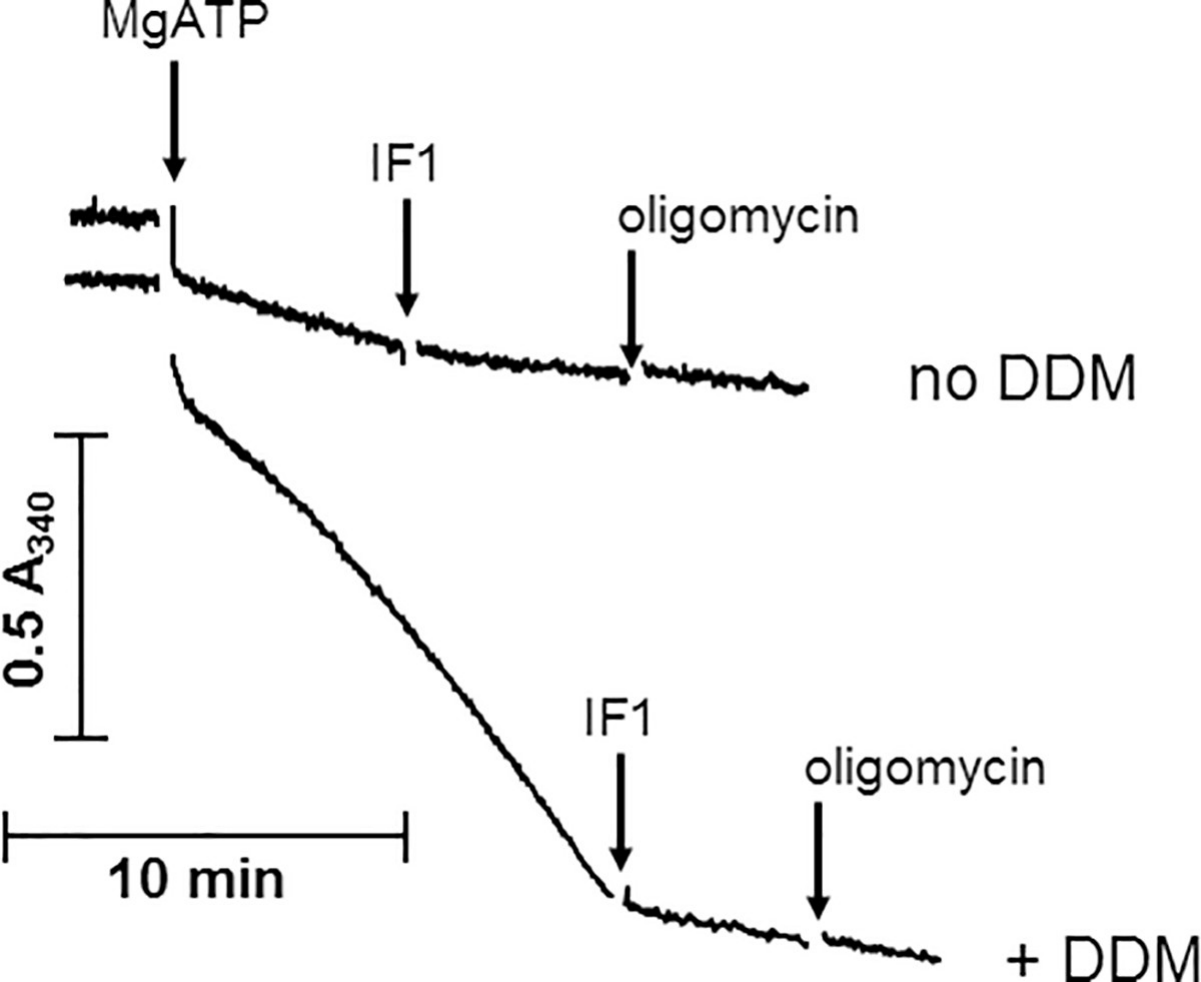
Time-course of ATP hydrolysis by solubilized fraction from murine brain, with and without DDM. Conditions as described under Materials and Methods; 13 μg protein content; vertical arrows = additions (1 mM MgATP, 3 μM IF1, 6 μM oligomycin). Total complex V activity is given by the steady state rate of ATP hydrolysis corrected from the final rate value after addition of IF1 + oligomycin and is expressed as nmol ATP hydrolyzed per min and per mg of protein. It was 168 in the absence of DDM and 925 in its presence.

The second relevant kinetic parameter was the catalytic constant k_cat_, also called turnover rate. It could not be inferred from the specific activity because the latter also depended on the proportion of complex V in the total protein amount. To compare k_cat_ values in the different murine tissues, we measured the complex V activity in mitochondria solubilized in mild detergent suitable for the evaluation of complex V amount using BN-PAGE Western blot analysis. The time course of ATP hydrolysis by such extracts differed from that previously observed on homogenates (Fig 4 and S9 Fig). In the absence of DDM in the assay medium, the activity was very low. In the presence of DDM, it was initially higher than in its absence and still increased with time, reaching a maximal value after more than 10 minutes. Disruption of intact mitochondria could not explain that stimulating effect of DDM, because the extracts did not contain membranes anymore. Moreover, it was much slower than the effect observed with homogenates (Fig 2). Incubating the extract with DDM for 20 min before MgATP addition allowed the activity to reach its maximal value immediately after substrate addition (see example in S9 Fig). Therefore, the filling of non-catalytic sites with ATP, a phenomenon widely observed with isolated F_1_-ATPase [20], could not explain the slow activation. We inferred that the extracts preparation in mild detergent produced a less than optimal functional state of complex V that required DDM for full activity recovery. We thus considered that the steady state IF1-sensitive rate of ATP hydrolysis in the presence of DDM was the relevant complex V activity.

To compare the k_cat_ value of complex V between two preparations, we submitted these preparations to BN-PAGE electrophoresis followed by Western blot using antibodies directed against the F_1_ domain of complex V. In each gel, we run a series of twofold dilutions of the two preparations, with the highest amount corresponding to 40 nanomoles per minute complex V activity, based on the previously measured steady state rate of ATP hydrolysis. Using a series of four twofold dilutions allowed addressing the linear relationship between signal intensity and amount deposited on the gel (S10 Fig). The correlation coefficient of the linear regression curve was above 0.9 for 18 of the 28 preparations analyzed. In the remaining preparations, withdrawal of one sample out of four restored linearity with a correlation coefficient above 0.9 in eight case, and at 0.85 and 0.81 in the two last preparations. The sample diverging from linearity was always the sample with the highest ATPase activity and its signal was always below the expected value showing that our experimental conditions could be limiting with the most concentrated samples. Insufficient Coomassie blue for proper sample separation, insufficient transfer, or inappropriate antigen/antibody ratio could all explain the observed discrepancy. Altogether, only 10 samples among 112 diverged from linearity. However, for the sake of consistency, we decided to systematically remove all the data with the highest ATPase activity (40 nmol min^-1^).

**Fig 5:**
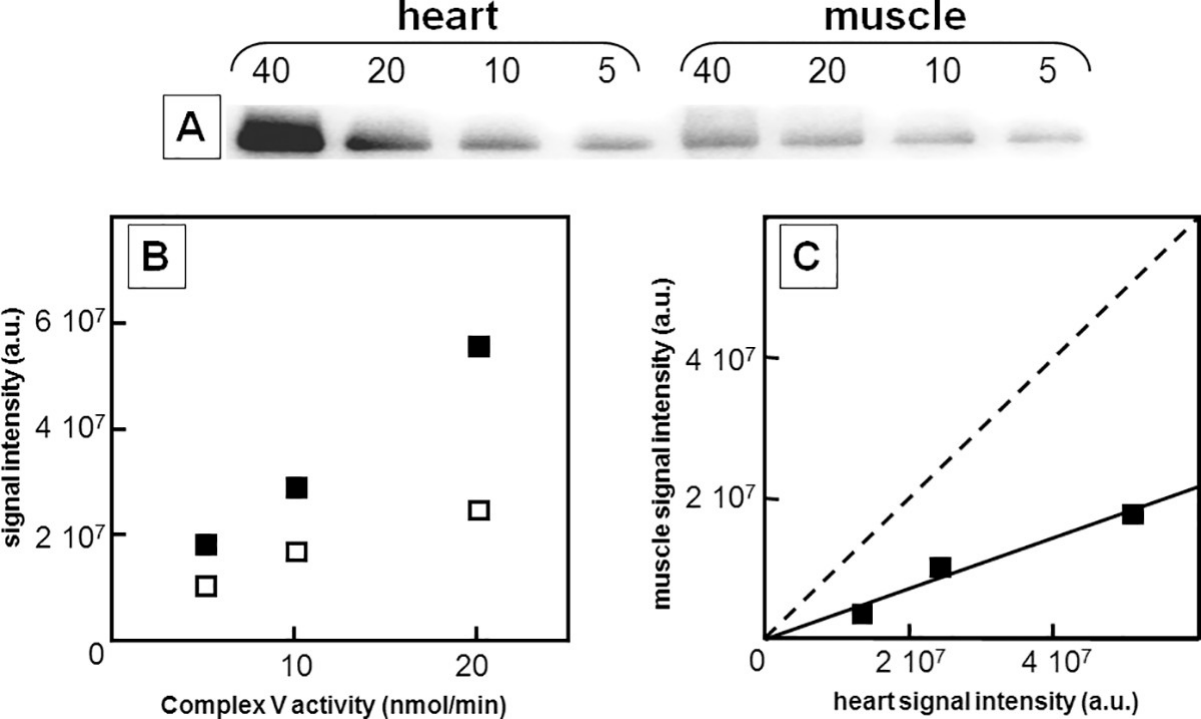
Immunotitration of complex V in solubilized fractions from heart and muscle homogenates. Conditions as described under Materials and Methods; panel A: complex V immunoblot obtained with one heart preparation and one muscle preparation; numbers above the lanes are the activity of the samples expressed as nmoles ATP hydrolyzed per minute. Panel B: densitometric signal intensity expressed in arbitrary units plotted against complex V activity; (■) heart, (□) muscle. Panel C: for each complex V activity, signal intensity from muscle preparation plotted against signal intensity from heart preparation (data drawn from results in Panel B); diagonal = dashed straight line. The slope of the graph (= 0.36) gives the ratio of complex V content between muscle and heart preparations at a given enzyme activity.

For each lane corresponding to the same complex V activity, we expected similar signal intensity if complex V had the same k_cat_ value in the two preparations. Fig 5 shows an example in which the gel contained one preparation from heart and one preparation from muscle. Plotting the intensity of the spots against the loaded activity (Fig 5 panel B) showed higher protein amount in heart than in muscle. Plotting, for each loaded activity, the intensity of the signal for muscle samples against that for heart samples gave a linear relationship with a slope around 0.36, clearly below the graph diagonal expected if, for a given enzymatic activity, the complex V amount were similar in muscle and heart samples.

**Fig 6:**
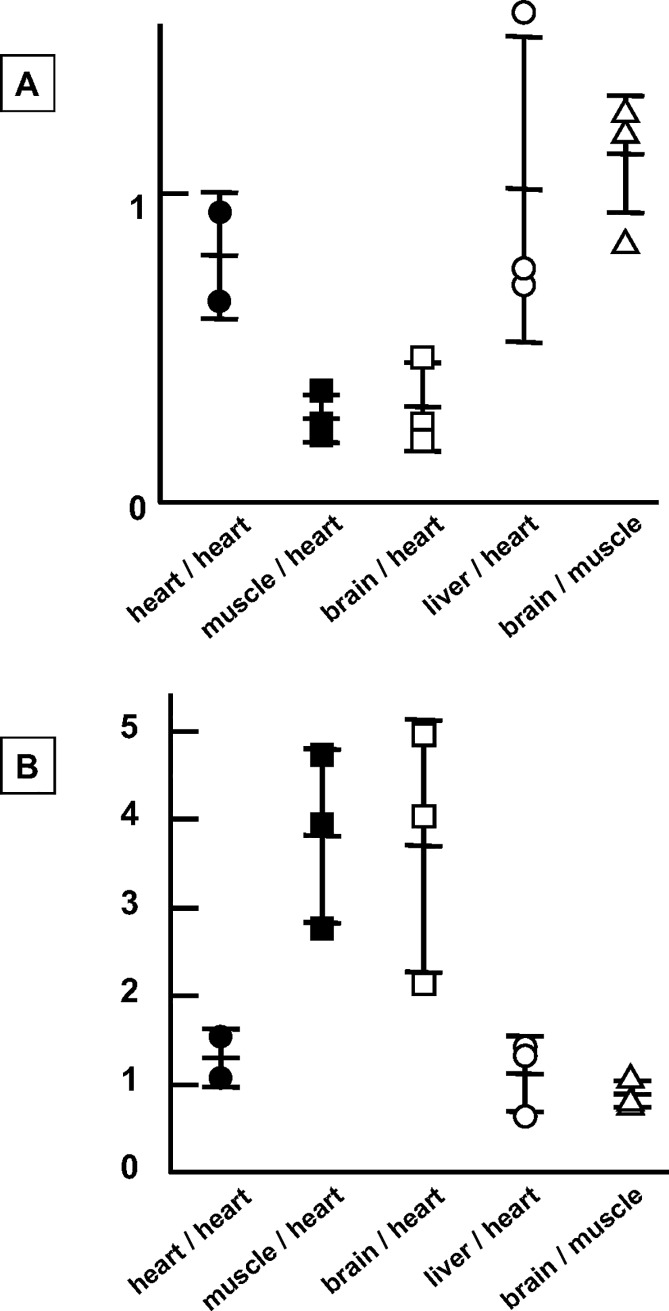
Ratio of complex V content between solubilized fractions from different organs at a given enzyme activity. Conditions as in Fig 5; Panel A: complex V ratio given by the slope of plots as described in Fig 5-panel C; Panel B: reciprocal of the complex V ratio, giving the ratio of complex V catalytic turnover values between the different organs. Individual values are indicated by the symbols while the lines draw the mean and standard deviation.

Fig 6 shows the result of 14 experiments like that displayed in Fig 5. Values reported in Panel A are the ratio of complex V amount found at a given ATPase activity between preparations of different organs and a single reference heart preparation (all bars except the right one), or between brain and muscle preparations (right bar). As expected, the complex V ratio found between two different heart preparations and the reference heart preparation was not very different from 1 (average: 0.80). This was also the case for the ratio between liver and heart (1.02 ± 0.50), but not for the ratios between muscle and heart (0.28 ± 0.08) and between brain and heart (0.31 ±14). Overall, this means that complex V had about the same k_cat_ value in heart and liver, and a higher k_cat_ value in muscle and brain since in the two latter cases a lower complex V amount was required to have a given ATPase activity. The k_cat_ values appeared similar in muscle and brain, as confirmed by the complex V ratio between brain and muscle (right bar, 0.99 ± 0.19). Panel B of Fig 6 shows the reciprocal of the complex V ratios displayed in Panel A. The values then directly show the complex V k_cat_ ratios between preparations of different organs. As previously mentioned, the complex V k_cat_ values were similar for heart and liver. They were similar for muscle and brain and reached more than 300% the k_cat_ value found for heart.

### Relative activities of complex I, II and V

To evaluate complex V activity with respect to that of respiratory chain, we investigated the activity of complex I and complex II in homogenates from frozen-thawed fragments of murine brain, muscle, liver and heart. To ensure experimental conditions as close as possible to those used for complex V activity measurements, 0.01% DDM was present in the reaction medium. These assays thus slightly differed from standardized assays routinely used in clinical diagnosis [21]. In complex I assay, samples from the different organs supplemented with decyl-ubiquinone presented with an unstable NADH oxidation activity that progressively decayed to a level close to zero (S11 Fig). Addition of a new sample restored an activity similar to that initially observed, which again decreased with time. The decay of activity was thus due to enzyme deactivation and not to the variation of substrates concentration. Fitting the first three minutes of the kinetics with a model of monoexponential decay of the activity allowed extrapolating the initial rate of NADH oxidation. Some decrease of absorbance at 340 nm also occurred in the presence of rotenone, KCN and antimycin and in the absence of decyl-ubiquinone. Therefore, it was not due to complex I. Its initial rate was calculated with the same fitting as before (dashed lines in S11 Fig) and subtracted to that previously estimated to obtain the specific complex I activity.

In the same samples preparations from the four organs, complex II activity was stable (S12 Fig). As expected, malonate addition completely inhibited the activity.

**Fig 7:**
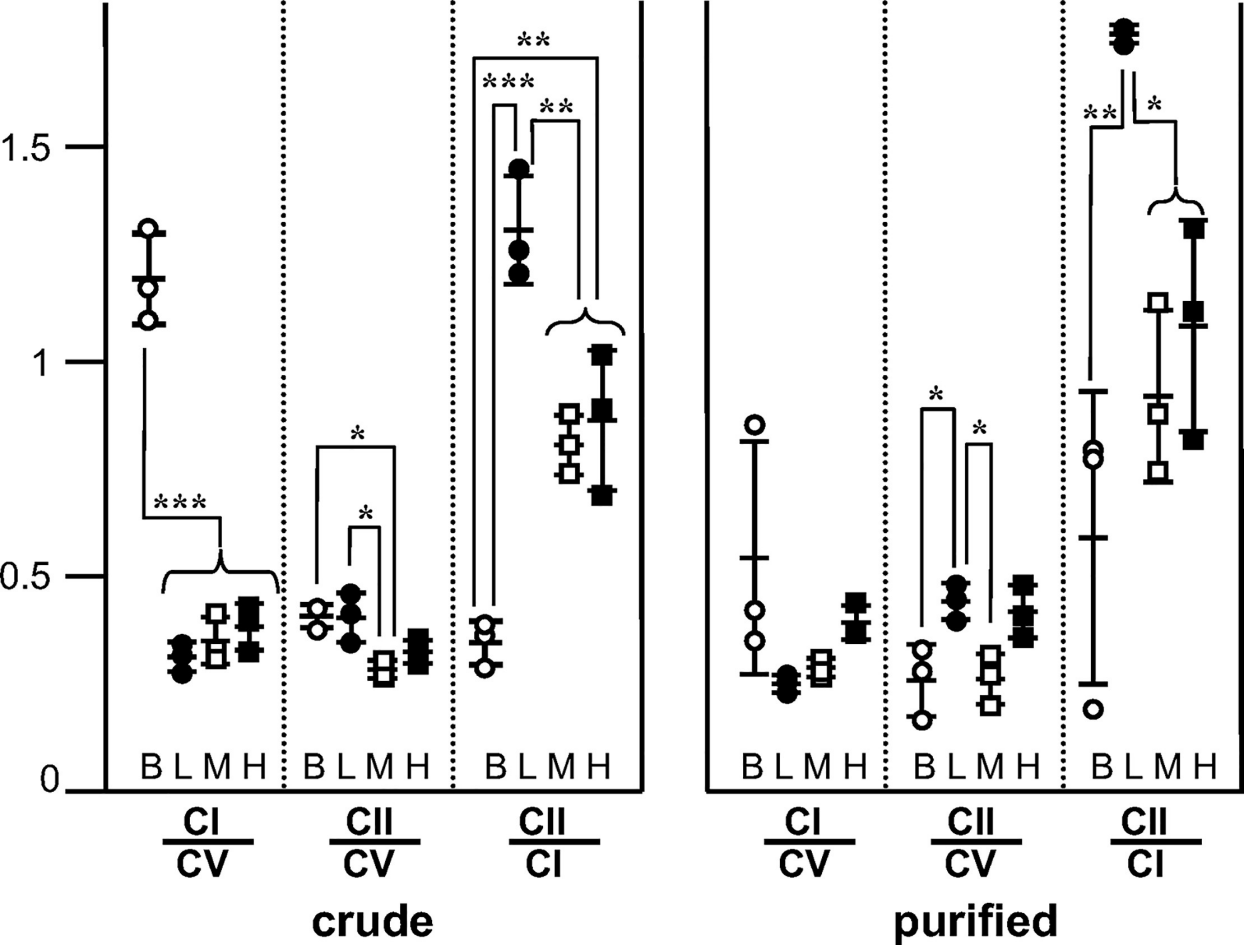
Relative activities of complex I, complex II and complex V in homogenates and mitochondrial pellet from brain, liver, muscle and heart. Conditions as described under Materials and Methods and in Fig 2; crude = supernatant obtained after low speed centrifugation of tissue homogenate; purified = pellet obtained after centrifuging the crude material at high speed; B = brain; L = Liver; M = muscle; H = heart; CI = complex I; CII = complex II; CV = complex V; CI/CV, CII/CV and CII/CI = ratios between the activities of the different complexes; individual symbols represent the result from independent frozen-thawed preparations of each organ, they consist of the average of two measurements of each activity; statistical significance * p<0.05, ** p<0.01, *** p<0.001.

We measured the activity of complex I, complex II and complex V in three different preparations from frozen-thawed fragments of each organ, each measured twice. From these data, we calculated the mean ratio between the different activities (Fig 7). The CI/CV ratio was comparable in preparations from liver, muscle and heart, but was much higher in those from brain. The CII/CV ratio was slightly higher in preparations from brain or liver than in those from muscle or heart. A higher ratio complex II/complex V in liver as compared to muscle and heart has already been reported using BN PAGE and human tissues [22]. The CII/CI ratio in liver, muscle and heart more or less followed the CII/CV ratio. It was very low in brain because of the very high CI/CV ratio. Taken together, these data suggested that the relative proportion of CV activity was similar in the diverse organs while CII and CI activity varied, CII activity being the highest in brain and liver and the lowest in muscle and CI being very high in brain as compared to other tissues. Crude homogenate preparations may contain some contaminants, including plasma membranes, which might contain complex V ATPase activity [23]. We thus investigated the relative activities of complex I, II and V in the pellet obtained after high-speed centrifugation of the crude material (11000 *g* during 10 minutes), which eliminated plasma membranes (Fig 7). Comparison of the different activities found in this purified preparation with those in the crude material showed a decrease of the CI/CV activity ratio in brain and, to a lesser extent, in liver and muscle. In contrast, CII/CV activity ratio decreased in brain and did not significantly vary in the other cases. Therefore, whatever the tissue, there was no evidence for complex V activity in plasma membranes, *i*.*e*. ectopic F_1_-ATPase, because, if that were the case, CI/CV and CII/CV activity ratios would have increased in purified preparations.

Since essentially brain showed the relative activity changes after purification, we investigated a possible degradation of activities by the centrifugation alone in the four different tissues. Centrifuging the crude homogenate preparations at high speed, followed by remixing pellet and supernatant, kept together all the components of the preparation. However, in brain, the activity of complex I, II and V decreased by about 55%, 45% and 20%, respectively, with respect to the initial value in crude homogenate. This was not the case for the other three tissues where degradation did not exceed 20%, whatever the complex. Selective degradation of the complexes thus probably occurred, especially in brain preparations, during high-speed centrifugation and/or re-homogenization of the pellet. That degradation explained the drop of CI/CV and CII/CV ratios in brain mitochondrial pellet as compared to homogenate. In conclusion, crude homogenate preparations, where no such a degradation occurs, gave a better picture of mitochondrial activities than the purified mitochondrial pellets, especially in brain. Homogenates were similarly considered relevant for the study of respiratory control and protonmotive force in rat tissues [24].

### Complex V activity in human muscle biopsies

To check the suitability of our method to human biopsies, we performed the assay in frozen biopsies from control muscles and from two patients with confirmed deleterious mutations of the *MT-ATP6* genes. Kinetics of ATP hydrolysis in the presence of DDM were similar to those obtained with murine tissues (see example in S13 Fig). The complex V activity of five controls, each one measured four times, was 266 ± 100 nmol ATP hydrolyzed per min and per mg of protein (Table 3). Based on the inhibitory effect of IF1 + oligomycin, complex V activity represented 73 ± 15%of the total ATPase activity. These values were similar to those obtained with murine muscle (Table 1). Therefore, the method developed with murine tissues appears suitable for human biopsies. We then measured complex V activity in the muscle from two patients with confirmed deleterious mutation of the *MT-ATP6* gene. Patient P1 has been previously reported as Patient 7 in [25]. At 26 years of age, he presented with episodic paralysis accesses and pes cavus. His blood lactate level was normal. He carried an apparently homoplasmic m.9176T>C p.Leu217Pro mutation in blood and muscle. Patient P2 presented with psychomotor retardation in childhood and ataxia occurring in late teenage. Sensorimotor peripheral neuropathy, cerebellar syndrome, pyramidal syndrome and mild elevation of lactate level in blood and cerebrospinal fluid were present at the time of the muscle biopsy. P2 carried an apparently homoplasmic m.9035T>C p. Leu170Pro mutation in blood and muscle [26, 27]. Although the number of control muscles is still insufficient to provide a reliable normal data set, the two patients had a complex V activity and a sensitivity to inhibitors that were below the lowest control value. Furthermore, their residual complex V activity was congruent with their clinical phenotype: P1 having a clearly milder phenotype than P2.

**Table 3:** Complex V activity in human muscle.

Sample	Complex V activity	Sensitivity to OM	Sensitivity to IF1	Sensitivity to IF1 + OM
CTRL 1 (50 y)	333 ± 42 (n = 4)	88%	88%	88 ± 1%
CTRL 2 (60 y)	367 ± 32 (n = 4)	78%	87%	88 ± 1%
CTRL 3 (56 y)	180 ± 31 (n = 4)	50%	56%	57 ± 5%
CTRL 4 (38 y)	355 ± 14 (n = 4)	70%	69%	75 ± 4%
CTRL 5 (72 y)	136 ± 15 (n = 4)	57%	48%	59 ± 6%
P1 *MT-ATP6* p.Leu217Pro (26 y)	122 ± 20 (n = 4)	13%	27%	28 ± 4%
P2 *MT-ATP6* p.Leu170Pro (29 y)	46 ± 5 (n = 4)	5%	10%	13 ± 1%

Sample = post nuclear supernatant from frozen-thawed muscle fragment sobtained during an orthopedic surgical procedure of patients without any sign suggesting a genetic disease (CTRL) or during an open muscle biopsy performed as a diagnostic investigation for a suspected mitochondrial disease (P1 and P2); the age at muscle sampling is indicated between brackets, y = years of age; Complex V activity = ATP hydrolysis activity sensitive to the combination of IF1 and oligomycin (OM), the mean and standard deviation of the activity are expressed as nanomoles ATP hydrolyzed per minute and milligram protein; n = number of assays; sensitivity to OM or to IF1 alone was evaluated by adding the inhibitor first; sensitivity to IF1 + OM was evaluated after addition of the second inhibitor; these sensitivities are expressed as % of the total hydrolysis activity; only sensitivity to IF1 + oligomycin is expressed as mean ± standard deviation of 4 assays while the sensitivity to each individual inhibitor is expressed as the mean of two measurements.

## Discussion

### On the interest of improving measurement of ATP hydrolysis

We addressed the functional study of complex V in different tissues in order to allow functional evaluation of mutations involving complex V genes but also analysis of expression regulation, post-translational modification or other processes on complex V capacity.

We measured ATP hydrolysis *i*.*e*. the reaction reverse to the physiologically relevant reaction catalyzed by complex V. Measurement of ATP synthesis is possible in isolated mitochondria or in permeabilized cells but requires fresh tissues that are rarely available in clinical diagnosis. Furthermore, it depends on numerous factors additional to complex V capacity such as the membrane H^+^-conductance and the activity of diverse enzymes including the redox carriers, the adenine nucleotide translocase and the phosphate transporter. Measurement of ATP synthesis will thus likely overlook subtle modifications of complex V function. On the other hand, the maximal ATPase activity of complex V, as estimated in this work, might differ from the maximal rate of ATP synthesis and thus might lead to misdiagnosing a complex V defect. However, in the rare cases where both rates were measured [13], the maximal rate of ATP synthesis was found comparable to the maximal rate of ATP hydrolysis. Decreased complex V-related ATP hydrolysis is observed in severe cases with decreased amount of complex V such as, for example, patients with mutations in the *TMEM* gene [7]. Our finding of low complex V-related ATP hydrolysis in the muscle of two adult patients with homoplasmic confirmed deleterious mutations of the *MT-ATP* gene demonstrated the relevance of the spectrophotometric assay for these relatively mild cases.

Alternative tissue disruption, using either osmotic choc or sonication, has been reported for the assay of ATP hydrolysis by complex V in fibroblasts and tissue homogenates [28–30]. In the present work, we used high pH and disrupting mitochondria with DDM because we thought that method easier to standardize than sonication. In isolated yeast mitochondria, alamethicin previously achieved membrane permeabilization [31]. This approach failed with homogenized tissues, in which we could not obtain reproducible results. We demonstrate here that gentle membrane solubilization by 0.01% DDM is an efficient alternative, allowing dilution of endogenous IF1 to non-inhibitory concentration as well as inhibition by added exogenous IF1 at a high concentration [31]. Disrupting mitochondrial membranes also allowed bypassing ATP/ADP exchange and phosphate translocation across the inner membrane, two processes that might kinetically limit complex V activity in intact mitochondria. Indeed, in mitochondria prepared from a *S*. *cerevisiae* mutant devoid of IF1, the rate of ATP hydrolysis measured in the presence of FCCP increased ten-fold after inner membrane permeabilization [31]. Here, based on the resistance to external IF1 addition (Fig 3), the activity of complex V in intact mitochondria was roughly 11% of that in disrupted mitochondria.

In conclusion, mitochondrial membrane permeabilization or disruption is quite important to insure measurement of maximal complex V activity.

### On the interest of using two different complex V inhibitors

In a biological preparation containing many ATPases, the activity of complex V is generally estimated by subtracting the ATPase activity resistant to oligomycin from the total activity. However, resistance to oligomycin can be due to functional uncoupling between F_0_ and F_1_ domains of complex V as well as to contaminating ATPase activities. To differentiate these two effects, we used IF1, an inhibitor directed to F_1_, the catalytic domain of complex V. IF1, added at high concentration (3 μM) before oligomycin, inhibited the complex V activity practically to the extent obtained with both oligomycin and IF1 (Fig 2). Conversely, adding oligomycin before IF1 showed that oligomycin alone inhibited activity to the extent obtained with both oligomycin and IF1 (see S5 Fig)). We thus demonstrated constant tight coupling between F_0_ and F_1_ domains, even in the case of brain homogenates where the oligomycin-resistant ATPase activity represented about 30% of the total activity. This is important in the context of pathological mutations where checking the integrity of proton coupling by complex V may be relevant. In this work, we also confirmed that yeast IF1 fully inhibited mammalian complex V [31, 32]. It could thus be used as well as mammalian IF1 in the analysis of mammalian complex V (S9 Fig).

### Tissue specificity of complex V catalytic mechanism

In this work, we report the first comparison of complex V catalytic properties in different tissues. Our results suggested that the catalytic turnover rate (k_cat_) of complex V was higher in brain and muscle than in heart and liver, whereas the Michaelis constant (K_m_) for MgATP was similar in the four tissues. It is difficult to predict how the k_cat_ differences observed on the enzyme towards ATP hydrolysis can influence cellular ATP synthesis. As discussed above, cellular mitochondrial ATP synthesis depends on many parameters, including the amount of mitochondria in the cell and the amount of complex V in mitochondria. We found that the average complex V specific activity in brain, liver and muscle homogenates was 7%, 20% and 25% respectively of the complex V specific activity in heart homogenate. Taking into account the relative k_cat_ values, the amount of complex V in these three homogenates would be respectively 2%, 19% and 7% of its value in heart. Accordingly, very low amount of complex V in brain has previously been reported using western blot of tissue homogenates [33].

Since there are no known isoforms of the subunits belonging to the catalytic F_1_-ATPase domain in mouse, the most plausible explanation for the tissue-dependent catalytic properties of complex V is post-translational modification(s). Tyrosine phosphorylation of the α subunit of complex V was reported in murine brain, but not in liver, muscle or heart [34]. However, that pattern does not match the k_cat_ tissue-specificity that we observed, showing that different post-translational modification(s) is(are) probably involved here.

### Relative activity of complexes I, II and V

We have measured the relative activities of complexes I, II and V in homogenates from different organs, in conditions that gave access to their maximal enzymatic capacity (Fig 7). The most striking finding was the very high relative activity of complex I in brain with respect to other organs, but the physiological meaning of this specificity is unknown. In addition, the relative activity of complex II was somewhat lower in muscle and heart than in brain and liver.

For a better comparison of the different complexes activities and their possible rate-limiting character in oxidative phosphorylation, it seems convenient to consider proton transport, which is common to the three complexes. In the redox chain, oxidation of one NADH molecule by oxygen results in the transport of 10 protons while oxidation of one succinate molecule results in the transport of six protons. In complex V, hydrolysis of one ATP molecule results in the transport of 2.66 protons. Taking into account these stoichiometries and the activity ratios that we observed (Fig 7), we can calculate that the proton transport capacity when entering through complex I is about 4.5 fold that of complex V in brain, 1.3 fold in liver, and 1.4 in muscle and heart. When entering through complex II, it is about 0.9 fold that of complex V in brain and liver, 0.6 fold in muscle and 0.7 fold in heart. Therefore, the proton transport capacities of complex I and II roughly equal that of complex V in muscle, heart and liver. In contrast, the complex I capacity seems oversized in brain. However, the respiratory rate coupled to ATP synthesis was at least as sensitive to complex I inhibition in rat mitochondria purified from brain as in mitochondria prepared from other organs while the observed excess of complex I activity would have implied the reverse [35]. Our present work suggests that this discrepancy is due to a loss of complex I activity during the preparation of brain mitochondria. Another possibility could be different tissue-dependency in rat and mouse.

### Ectopic F_1_-ATPases

Many reports have mentioned mitochondrial-type ATPases bound to the plasma membrane of different cells, with the catalytic part F_1_ facing the outer medium: the so-called “ectopic F_0_F_1_-ATPases” (for a review [36]). Due to their orientation with respect to the polarization of plasma membrane, ectopic F_0_F_1_-ATPases probably do not synthesize ATP under physiological conditions. However, they readily hydrolyze ATP and are inhibited by oligomycin or IF1. For this reason, one may wonder to which extent they could contribute to the complex V activity measured in our experiments. We observed that F_0_F_1_-ATPase activity in intact mitochondria was about 10% of the maximal activity (Fig 3). If complex V were fully inactive in intact mitochondria, that percentage could as well represent ectopic ATPase activity. This 10% value would then be an upper limit for the contribution of ectopic F_0_F_1_-ATPases. Biochemical analysis estimated the ectopic F_0_F_1_-ATPase in rat liver as about 5% of complex V amount [23]. It seems therefore reasonable to think that the weight of ectopic F_0_F_1_-ATPase, if any, is almost negligible in our experiments. This view was strengthened by the full elimination of the plasma membrane, which is not believed to contain complex I and complex II, not resulting in a decrease of complex V activity with respect to complex I and complex II activities (Fig 7).

### Perspectives

As compared to previous approaches using purified mitochondria [37–39], the main interest of the present method is to allow a fast, simple and reliable estimate of the complex V activity and of the integrity of functional coupling between F_1_ and F_0_ in a tissue crude homogenate. The only required equipment for the preparation is a small Potter tube and a benchtop centrifuge. The full preparation takes about half an hour and uses only some milligrams of biological material. Importantly, it gives similar rates of ATP hydrolysis with fresh or frozen tissues. Preparations are functionally stable for 1–2 days, which allows deep analyses, such as estimations of different respiratory complexes activity or detailed kinetic studies of complex V. Finally, the method proved to be suitable using human biopsies from muscle, making it a useful procedure for probing complex V functional properties in human biopsies in normal or pathological context.

## Supporting information

S1 Fig Schematic representation of the spectrophotometric assay for F1F0 ATP synthase activity. In physiological conditions, F1F0 ATP synthase catalyzes the phosphorylation of ADP into ATP. The reaction is reversible. The spectrophotometric assay measures the reverse reaction i.e. ATP hydrolysis into ADP. In the presence of pyruvate kinase in excess, the formed ADP is used to synthetize pyruvate from phosphoenolpyruvate and ATP is regenerated. Lactate dehydrogenase, present with NADH, both in excess, catalyzes the reduction of pyruvate into lactate and the oxidation of NADH into NAD.
The kinetics of the absorbance at 340 nm, specific for NADH, thus measures the rate of ATP hydrolysis. ATP concentration remains constant during all the kinetics.
Two inhibitors prevent alternative substrate utilization. Ap5A inhibits adenylate kinase, which catalyzes the reaction 2 ADP—> AMP + ATP. Antimycin A, an inhibitor of complex III, blocks electron transfer in the respiratory chain and, thus, the oxidation of NADH by complex I of the respiratory chain.
Oligomycin and Inhibitory Factor 1 (IF1) are two specific F1F0 ATP synthase, blocking the F0 and F1 domains respectively. They allow measuring the contribution of F1F0 ATP synthase to the observed ATP hydrolysis
(DOCX)

S2 Fig Estimation of endogenous ATP and ADP by rate extrapolation. Conditions as described under Materials and Methods; 8 μg homogenate of frozen-thawed tissue from heart; rate of ATP hydrolysis sensitive to IF1 + oligomycin is expressed in absorbance units per minute.
Panel A: rate of ATP hydrolysis as a function of the concentration of added MgATP; Panel B: data from Panel A restricted to the linear part of the plot. Note that the rate is not null in the absence of added MgATP, which indicates the presence of endogenous ATP (if present, ADP is transformed into ATP by the pyruvate kinase / lactate dehydrogenase regenerating system). The negative intercept of the regression line with X-axis gives the opposite value to be added to MgATP concentrations to obtain a null reaction rate at zero MgATP. This correction represents endogenous MgATP concentration in the cuvette: here 2.3 μM. Panel C, rate of ATP hydrolysis as a function of crude (□) and corrected (■) MgATP concentration.
(DOCX)

S3 Fig Sequence of inhibitory peptides IF1 from different species. The partial sequence from *Bos taurus* written in bold (a) is sufficient for fully preserving the inhibitory effect of IF1 and its high affinity for F_1_-ATPase [40]. The peptide with the partial sequence from *Saccharomyces cerevisiae* in bold (d) inhibits F_0_F_1_ ATPase activity of murine tissue homogenates with the same efficiency as the full peptide (this work). Replacement of the underlined residue (F28) by a tryptophan increased the peptide absorbance at 280 nm and facilitated its purification when overexpressed in *E*. *coli*. This mutation did not alter its inhibitory properties [17].
(DOCX)

S4 Fig Time-course of ATP hydrolysis by homogenates from different frozen-thawed organs. Conditions as described under Materials and Methods; 0.01% DDM; vertical lines = additions (1 mM MgATP, 3 μM IF1, 6 μM oligomycin); protein content = 32 μg for brain, 42 μg for liver, 50 μg for muscle, 13 μg for heart. The initial absorbance drop after MgATP addition is due to the consumption of some contaminating ADP. Specific activity, sensitive to IF1 and oligomycin, expressed as nmol ATP hydrolyzed per min and per mg protein, were: 103 for brain, 353 for liver, 269 for muscle, and 1479 for heart; it represented 79% (brain), 90% (liver), 95% (muscle), and 96% (heart) of the crude activity.
(DOCX)

S5 Fig Tight coupling between F_0_ and F_1_ domains of complex V. Conditions as described under Materials and Methods; 0.01% DDM; homogenates of frozen-thawed muscle and brain; addition of complex V inhibitors were switched.
Whether IF1, a F_1_ domain inhibitor, or oligomycin, a F_0_ domain inhibitor, was first added, the inhibition was similar to that obtained with the combination of IF1 + oligomycin. The presence of DDM thus did not impair the functional coupling between F_1_ and F_0_ domains.
(DOCX)

S6 Fig Initial contaminating activity observed with some brain homogenates. Conditions as described under Materials and Methods; 0.01% DDM; homogenates of frozen-thawed brain.
Left, kinetics of absorbance decrease at 340 nm. The absorbance rate slowly decays from an initially high value to a steady state value. The steady state activity is sensitive to IF1 and oligomycin additions.
Right, kinetics observed after 5 min pre-incubation of the sample in the presence (upper trace) or in the absence (middle trace) of IF1 and oligomycin. The asymptotes of these two curves give steady state activities expressed as absorbance units per minute. The difference between the two kinetics is displayed below the two traces; it shows the fraction of the activity sensitive to IF1 + oligomycin; the slope of the curve is constant and gives an estimated activity close to the difference of steady state activities without and with F_1_F_0_ inhibitors. This experience shows that the initial transient activity is not related to F_1_F_0_.
(DOCX)

S7 Fig Steady-state rate of ATP hydrolysis sensitive to (IF1 + oligomycin) as a function of the protein amount. Conditions as in S4 Fig; four homogenates of different frozen-thawed tissues from heart (●), liver (○), muscle (■) and brain (□); specific activities expressed as nmol ATP hydrolyzed per min and per mg protein were 1390 for heart, 427 for liver, 312 for muscle, and 100 for brain. Inset, 8-fold enlargement of brain plot along the Y-axis.
(DOCX)

S8 Fig Normalized rate of ATP hydrolysis sensitive to (IF1 + oligomycin) as a function of MgATP concentration. Conditions as in S4 Fig; homogenates of different frozen-thawed tissues from brain, liver, muscle and heart; each data point = average of two measurements; different symbols (■, □, ▲, Δ) = independent preparations (four for brain, two for liver and heart, three for muscle. We corrected MgATP concentrations for endogenous ATP as indicated in S3 Fig. For each preparation, we averaged two rate measurements performed at each MgATP concentration. Data were then fitted with the Michaelis-Menten equation. The rates at given MgATP concentrations were normalized to V_max_ for each preparation and the normalized values from the different preparations were merged and fitted again with the Michaelis-Menten equation; continuous line = data fitting with the mean computed value of K_m_; dashed lines = data fitting with the mean value of K_m_ plus or minus the standard deviation. Estimated Km values (μM): 148 ± 12 (brain); 220 ± 22 (liver); 217 ± 19 (muscle); 146 ± 11 μM (heart).
(DOCX)

S9 Fig Time-course of ATP hydrolysis in presence of DDM by solubilized fractions from different organs. Conditions as in Fig 4 (main text); vertical arrows = additions (1 mM MgATP, 3 μM IF1, 6 μM oligomycin). First panel: protein content: 13 μg for brain, 9.5 μg for liver, 1.5 μg for heart, and 1.4 μg for muscle. Complex V activity expressed as nmol ATP hydrolyzed per min and per mg of protein was 925 for brain, 1122 for liver, 3104 for heart and 2691 for muscle. Second panel: comparison of kinetics obtained by adding MgATP about 2 min after the sample (upper trace) or about 30 min after (lower trace). That experience shows that the activation process proceeds in the absence as well as in the presence of MgATP.
(DOCX)

S10 Fig Immunotitration of complex V in solubilized fractions from brain and muscle homogenates. Conditions as in Fig 5 (main text). Upper panel = complex V immunoblot with samples of brain and muscle preparations at given complex V activities indicated on top of the lanes and expressed as nanomoles/minute. Lower panel = linear regression analysis of the signal intensities expressed as arbitrary units (A.U.) plotted against complex V activity; black symbols = samples from muscle, white symbols = signals from brain. The signals corresponding to 40 nanomoles ATP hydrolyzed per minute (between parentheses) diverged from linearity, being lower than expected. The deviation was significant for brain samples whose correlation coefficient was 0.66 when taking into account the four data points of the series and 0.96 when eliminating the data point corresponding to the highest activity.
Linear regression analysis after withdrawal of the data points corresponding to the highest activity showed correlation coefficients close to 1 and a slope similar for brain and muscle preparations analyzed on the same gel.
(DOCX)

S11 Fig Time-course of complex I-dependent NADH oxidation by homogenates from different organs. Conditions as described under Materials and Methods; vertical arrows = sample (S) addition to the reaction medium; +10-UQ = presence of decyl-ubiquinone, a complex I substrate; no 10-UQ+rotenone = absence of the substrate and presence of rotenone, a complex I inhibitor; protein amount in the assay: 35 μg (brain), 47 μg (liver), 83 μg (muscle), 18 μg (heart). The dashed straight lines show the initial rates computed by fitting the first three minutes of the kinetics with a monoexponential curve. Complex I specific activity, expressed as nanomoles NADH oxidized per minute and per mg protein, was 169 for brain, 98 for liver, 168 for muscle, and 1164 for heart. NADH oxidation, unrelated to complex I, was 12% of the total activity for brain, 13% for liver, 1% for muscle, and 2% for heart.
(DOCX)

S12 Fig Time-course of complex II-dependent DCPIP reduction by homogenates from different organs. Conditions as described under Materials and Methods; vertical arrows = addition to the reaction medium of sample (S) or malonate, an inhibitor of complex II; protein amount in the assay: 108 μg (brain), 47 μg (liver), 83 μg (muscle), 9 μg (heart). Complex II specific activity, expressed as nanomoles NADH oxidized per minute and per mg protein, was 56 for brain, 137 for liver, 120 for muscle, and 847 for heart
(DOCX)

S13 Fig Time-course of complex V assay in human muscle. Conditions as described under Materials and Methods; vertical arrows = addition to the reaction medium of 1 mM MgATP, 3 μM IF1 or 6 μM oligomycin. ATP hydrolysis activity, expressed as nanomoles ATP hydrolyzed per minute and per mg protein, was 385 for a fragment of gluteus maximus (upper trace, 16 μg protein in the assay) and 354 for a fragment of gluteus minimus (lower trace, 34 μg protein in the assay). In both cases, IF1 + oligomycin inhibited 88% of the activity.
(DOCX)

## Abbreviations

IF1: Inhibitory factor 1
Pmf: protonmotive force
DDM: dodecylmaltoside
EDTA: **e**thylenediaminetetraacetic acid
BSA: bovine serum albumin
NADH: reduced nicotinamide adenine dinucleotide
DCPIP: 2,6-Dichlorophenolindophenol
KCN: potassium cyanide
SDS: sodium dodecylsulfate
