# Supplementary material for: Kinetic analysis of ATP hydrolysis by complex V in four murine tissues: Towards an assay suitable for clinical diagnosis
Source: PLoS One. 2019 Aug 28;14(8):e0221886. doi: 10.1371/journal.pone.0221886 (PMC6713359; doi:10.1371/journal.pone.0221886)
Supplement: S1 Fig — In physiological conditions, F1F0 ATP synthase catalyzes the phosphorylation of ADP into ATP. The reaction is reversible. The spectrophotometric assay measures the reverse reaction i.e. ATP hydrolysis into ADP. In the presence of pyruvate kinase in excess, the formed ADP is used to synthetize pyruvate from phosphoenolpyruvate and ATP is regenerated. Lactate dehydrogenase, present with NADH, both in excess, catalyzes the reduction of pyruvate into lactate and the oxidation of NADH into NAD. The kinetics of the absorbance at 340 nm, specific for NADH, thus measures the rate of ATP hydrolysis. ATP concentration remains constant during all the kinetics. Two inhibitors prevent alternative substrate utilization. Ap5A inhibits adenylate kinase, which catalyzes the reaction 2 ADP—> AMP + ATP. Antimycin A, an inhibitor of complex III, blocks electron transfer in the respiratory chain and, thus, the oxidation of NADH by complex I of the respiratory chain. Oligomycin and Inhibitory Factor 1 (IF1) are two specific F1F0 ATP synthase, blocking the F0 and F1 domains respectively. They allow measuring the contribution of F1F0 ATP synthase to the observed ATP hydrolysis (DOCX) [file pone.0221886.s001.docx]

ATP

***pyruvate kinase***

***lactate dehydrogenase***

NADH

NAD

lactate

Reading at 340 nm

***Added inhibitors:***

- Ap5A inhibits utilization of ADP by *adenylate kinase*
- Antimycin A inhibits oxidation of NADH by the respiratory chain

phosphoenolpyruvate

pyruvate

***F_1_F_0_ ATP synthase***

***complex V***

ATP + H2O

ADP

Phosphate

**S1 Fig. Schematic representation of the spectrophotometric assay for F_1_F_0_ ATP synthase activity**

In physiological conditions, F_1_F_0_ ATP synthase catalyzes the phosphorylation of ADP into ATP. The reaction is reversible. The spectrophotometric assay measures the reverse reaction i.e. ATP hydrolysis into ADP. In the presence of pyruvate kinase in excess, the formed ADP is used to synthetize pyruvate from phosphoenolpyruvate and ATP is regenerated. Lactate dehydrogenase, present with NADH, both in excess, catalyzes the reduction of pyruvate into lactate and the oxidation of NADH into NAD.

The kinetics of the absorbance at 340 nm, specific for NADH, thus measures the rate of ATP hydrolysis. ATP concentration remains constant during all the kinetics.

Two inhibitors prevent alternative substrate utilization. Ap5A inhibits adenylate kinase, which catalyzes the reaction 2 ADP AMP + ATP. Antimycin A, an inhibitor of complex III, blocks electron transfer in the respiratory chain and, thus, the oxidation of NADH by complex I of the respiratory chain.

Oligomycin and Inhibitory Factor 1 (IF1) are two specific F_1_F_0_ ATP synthase, blocking the F_0_ and F_1_ domains respectively. They allow measuring the contribution of F_1_F_0_ ATP synthase to the observed ATP hydrolysis.
