# Supplementary material for: Kinetic analysis of ATP hydrolysis by complex V in four murine tissues: Towards an assay suitable for clinical diagnosis
Source: PLoS One. 2019 Aug 28;14(8):e0221886. doi: 10.1371/journal.pone.0221886 (PMC6713359; doi:10.1371/journal.pone.0221886)
Supplement: S2 Fig — Conditions as described under Materials and Methods; 8 μg homogenate of frozen-thawed tissue from heart; rate of ATP hydrolysis sensitive to IF1 + oligomycin is expressed in absorbance units per minute. Panel A: rate of ATP hydrolysis as a function of the concentration of added MgATP; Panel B: data from Panel A restricted to the linear part of the plot. Note that the rate is not null in the absence of added MgATP, which indicates the presence of endogenous ATP (if present, ADP is transformed into ATP by the pyruvate kinase / lactate dehydrogenase regenerating system). The negative intercept of the regression line with X-axis gives the opposite value to be added to MgATP concentrations to obtain a null reaction rate at zero MgATP. This correction represents endogenous MgATP concentration in the cuvette: here 2.3 μM. Panel C, rate of ATP hydrolysis as a function of crude (□) and corrected (■) MgATP concentration. (DOCX) [file pone.0221886.s002.docx]

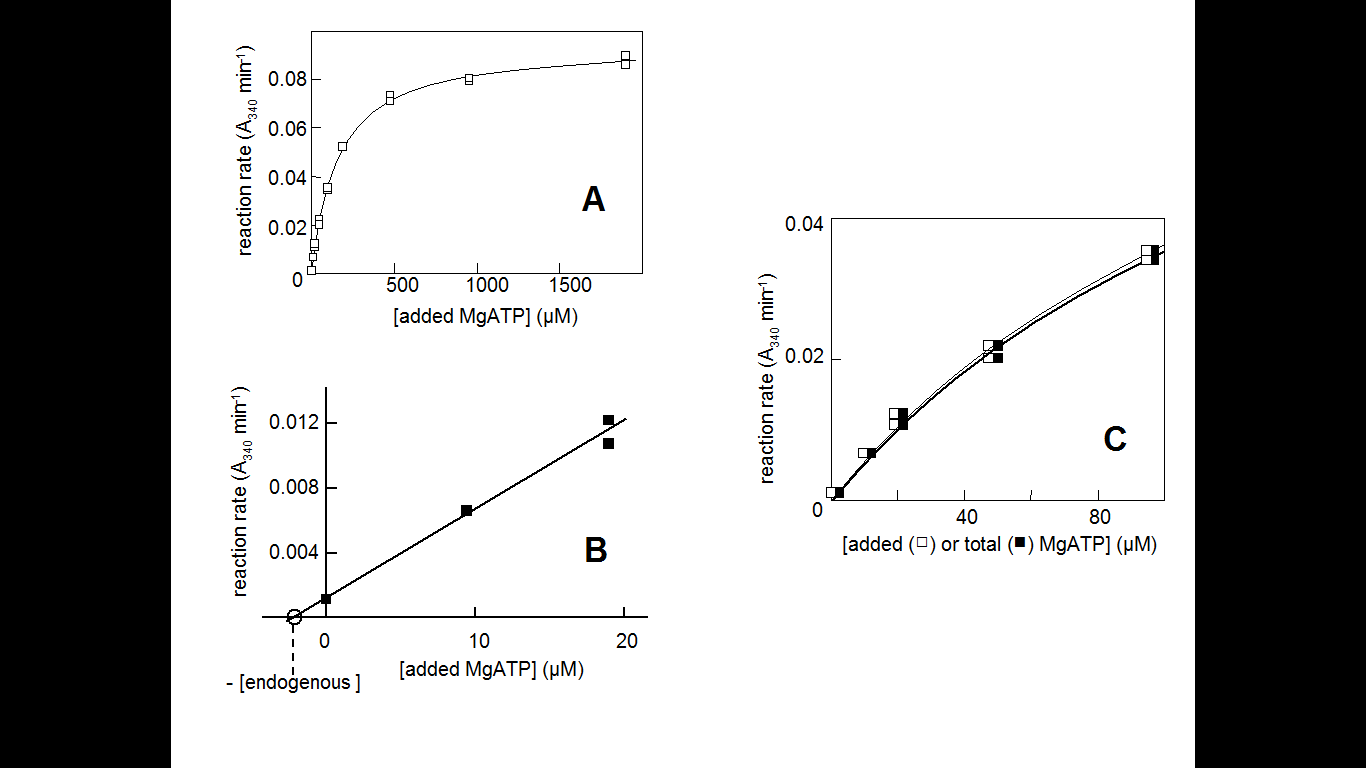


**S2 Fig. Estimation of endogenous ATP and ADP by rate extrapolation.**

Conditions as described under Materials and Methods; 8 µg homogenate of frozen-thawed tissue from heart; rate of ATP hydrolysis sensitive to IF1 + oligomycin is expressed in absorbance units per minute.

Panel A: rate of ATP hydrolysis as a function of the concentration of added MgATP; Panel B: data from Panel A restricted to the linear part of the plot. Note that the rate is not null in the absence of added MgATP, which indicates the presence of endogenous ATP (if present, ADP is transformed into ATP by the pyruvate kinase / lactate dehydrogenase regenerating system). The negative intercept of the regression line with X-axis gives the opposite value to be added to MgATP concentrations to obtain a null reaction rate at zero MgATP. This correction represents endogenous MgATP concentration in the cuvette: here 2.3 µM. Panel C, rate of ATP hydrolysis as a function of crude (□) and corrected (■) MgATP concentration.
