# Supplementary material for: Kinetic analysis of ATP hydrolysis by complex V in four murine tissues: Towards an assay suitable for clinical diagnosis
Source: PLoS One. 2019 Aug 28;14(8):e0221886. doi: 10.1371/journal.pone.0221886 (PMC6713359; doi:10.1371/journal.pone.0221886)
Supplement: S3 Fig — The partial sequence from Bos taurus written in bold (a) is sufficient for fully preserving the inhibitory effect of IF1 and its high affinity for F1-ATPase [40]. The peptide with the partial sequence from Saccharomyces cerevisiae in bold (d) inhibits F0F1 ATPase activity of murine tissue homogenates with the same efficiency as the full peptide (this work). Replacement of the underlined residue (F28) by a tryptophan increased the peptide absorbance at 280 nm and facilitated its purification when overexpressed in E. coli. This mutation did not alter its inhibitory properties [17]. (DOCX) [file pone.0221886.s003.docx]

a GSESGDNVR**S SAGAVRDAGG AFGKREQAEE ERYFRARAKE QLAALKKHHE NEISHH**AKEI ERLQKEIERH KQSIKKLKQS EDDD

b GSDQSENVDR GAGSIREAGG AFGKREQAEE ERYFRAQSRE QLAALKKHHE EEIVHHKKEI ERLQKEIERH KQKIKMLKH- -DDR

c VSDSSDSMDT GAGSIREAGG AFGKREKAEE DRYFREKTKE QLAALRKHHE DEIDHHSKEI ERLQKQIERH KKKIQQLKNN -H--

d **-SEGSTGTPR GSG----SED SFVKRERATE DFFVRQREKE QLRHLK---- EQLEKQRKKI DS**LENKIDSM TK-------- ----

a : *Bos taurus*

b : *Homo sapiens*

c : *Mus musculus*

d : *Saccharomyces cerevisiae*

**S3 Fig. Sequence of inhibitory peptides IF1 from different species**.

The partial sequence from *Bos taurus* written in bold (a) is sufficient for fully preserving the inhibitory effect of IF1 and its high affinity for F_1_-ATPase [1]. The peptide with the partial sequence from *Saccharomyces cerevisiae* in bold (d) inhibits F_0_F_1_ ATPase activity of murine tissue homogenates with the same efficiency as the full peptide (this work). Replacement of the underlined residue (F28) by a tryptophan increased the peptide absorbance at 280 nm and facilitated its purification when overexpressed in *E. coli*. This mutation did not alter its inhibitory properties [2].

1. van Raaij MJ, Orriss GL, Montgomery MG, Runswick MJ, Fearnley IM, Skehel JM, et al. The ATPase inhibitor protein from bovine heart mitochondria: the minimal inhibitory sequence. Biochemistry. 1996;35(49):15618-25. doi: 10.1021/bi960628f. PubMed PMID: 8961923.

2. Andrianaivomananjaona T, Moune-Dimala M, Herga S, David V, Haraux F. How the N-terminal extremity of Saccharomyces cerevisiae IF1 interacts with ATP synthase: a kinetic approach. Biochim Biophys Acta. 2011;1807(2):197-204. PubMed PMID: 20951672.
