# Supplementary material for: Kinetic analysis of ATP hydrolysis by complex V in four murine tissues: Towards an assay suitable for clinical diagnosis
Source: PLoS One. 2019 Aug 28;14(8):e0221886. doi: 10.1371/journal.pone.0221886 (PMC6713359; doi:10.1371/journal.pone.0221886)
Supplement: S4 Fig — Conditions as described under Materials and Methods; 0.01% DDM; vertical lines = additions (1 mM MgATP, 3 μM IF1, 6 μM oligomycin); protein content = 32 μg for brain, 42 μg for liver, 50 μg for muscle, 13 μg for heart. The initial absorbance drop after MgATP addition is due to the consumption of some contaminating ADP. Specific activity, sensitive to IF1 and oligomycin, expressed as nmol ATP hydrolyzed per min and per mg protein, were: 103 for brain, 353 for liver, 269 for muscle, and 1479 for heart; it represented 79% (brain), 90% (liver), 95% (muscle), and 96% (heart) of the crude activity. (DOCX) [file pone.0221886.s004.docx]

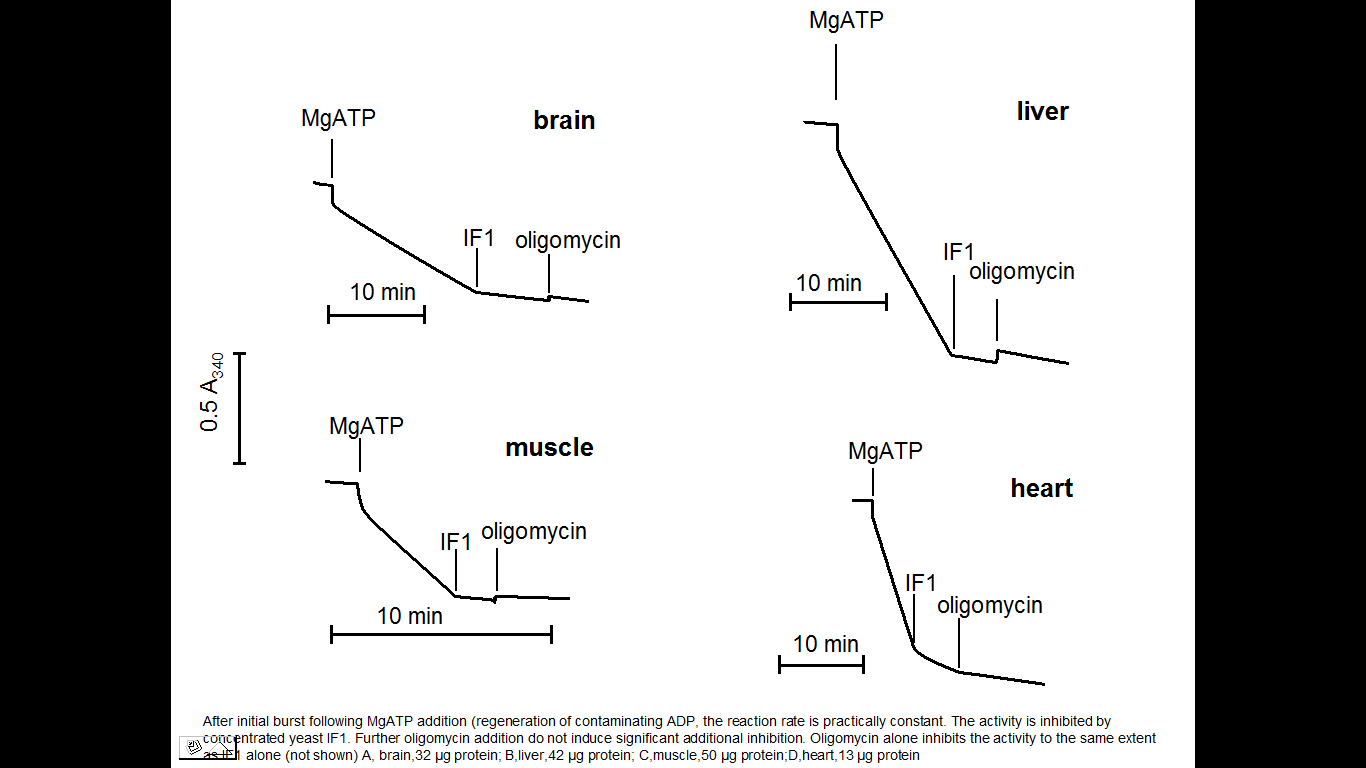


**S4 Fig. Time-course of ATP hydrolysis by homogenates from different frozen-thawed organs.**

Conditions as described under Materials and Methods; 0.01% DDM; vertical lines = additions (1 mM MgATP, 3 µM IF1, 6 µM oligomycin); protein content = 32 µg for brain, 42 µg for liver, 50 µg for muscle, 13 µg for heart. The initial absorbance drop after MgATP addition is due to the consumption of some contaminating ADP. Specific activity, sensitive to IF1 and oligomycin, expressed as nmol ATP hydrolyzed per min and per mg protein, were : 103 for brain, 353 for liver, 269 for muscle, and 1479 for heart; it represented 79 % (brain), 90% (liver), 95 % (muscle), and 96% (heart) of the crude activity.
