# Supplementary material for: Kinetic analysis of ATP hydrolysis by complex V in four murine tissues: Towards an assay suitable for clinical diagnosis
Source: PLoS One. 2019 Aug 28;14(8):e0221886. doi: 10.1371/journal.pone.0221886 (PMC6713359; doi:10.1371/journal.pone.0221886)
Supplement: S5 Fig — Conditions as described under Materials and Methods; 0.01% DDM; homogenates of frozen-thawed muscle and brain; addition of complex V inhibitors were switched. Whether IF1, a F1 domain inhibitor, or oligomycin, a F0 domain inhibitor, was first added, the inhibition was similar to that obtained with the combination of IF1 + oligomycin. The presence of DDM thus did not impair the functional coupling between F1 and F0 domains. (DOCX) [file pone.0221886.s005.docx]

**S5 Fig. Tight coupling between F_0_ and F_1_ domains of complex V.**

Conditions as described under Materials and Methods; 0.01% DDM; homogenates of frozen-thawed muscle and brain; addition of complex V inhibitors were switched.

Whether IF1, a F_1_ domain inhibitor, or oligomycin, a F_0_ domain inhibitor, was first added, the inhibition was similar to that obtained with the combination of IF1 + oligomycin. The presence of DDM thus did not impair the functional coupling between F_1_ and F_0_ domains.
