# Supplementary material for: Kinetic analysis of ATP hydrolysis by complex V in four murine tissues: Towards an assay suitable for clinical diagnosis
Source: PLoS One. 2019 Aug 28;14(8):e0221886. doi: 10.1371/journal.pone.0221886 (PMC6713359; doi:10.1371/journal.pone.0221886)
Supplement: S6 Fig — Conditions as described under Materials and Methods; 0.01% DDM; homogenates of frozen-thawed brain. Left, kinetics of absorbance decrease at 340 nm. The absorbance rate slowly decays from an initially high value to a steady state value. The steady state activity is sensitive to IF1 and oligomycin additions. Right, kinetics observed after 5 min pre-incubation of the sample in the presence (upper trace) or in the absence (middle trace) of IF1 and oligomycin. The asymptotes of these two curves give steady state activities expressed as absorbance units per minute. The difference between the two kinetics is displayed below the two traces; it shows the fraction of the activity sensitive to IF1 + oligomycin; the slope of the curve is constant and gives an estimated activity close to the difference of steady state activities without and with F1F0 inhibitors. This experience shows that the initial transient activity is not related to F1F0. (DOCX) [file pone.0221886.s006.docx]

**S6 Fig. Initial contaminating activity observed with some brain homogenates.**

Conditions as described under Materials and Methods; 0.01% DDM; homogenates of frozen-thawed brain.

Left, kinetics of absorbance decrease at 340 nm. The absorbance rate slowly decays from an initially high value to a steady state value. The steady state activity is sensitive to IF1 and oligomycin additions.

Right, kinetics observed after 5 min pre-incubation of the sample in the presence (upper trace) or in the absence (middle trace) of IF1 and oligomycin. The asymptotes of these two curves give steady state activities expressed as absorbance units per minute. The difference between the two kinetics is displayed below the two traces; it shows the fraction of the activity sensitive to IF1 + oligomycin; the slope of the curve is constant and gives an estimated activity close to the difference of steady state activities without and with F_1_F_0_ inhibitors. This experience shows that the initial transient activity is not related to F_1_F_0_.
