# Supplementary material for: Kinetic analysis of ATP hydrolysis by complex V in four murine tissues: Towards an assay suitable for clinical diagnosis
Source: PLoS One. 2019 Aug 28;14(8):e0221886. doi: 10.1371/journal.pone.0221886 (PMC6713359; doi:10.1371/journal.pone.0221886)
Supplement: S7 Fig — Conditions as in S4 Fig; four homogenates of different frozen-thawed tissues from heart (●), liver (○), muscle (■) and brain (□); specific activities expressed as nmol ATP hydrolyzed per min and per mg protein were 1390 for heart, 427 for liver, 312 for muscle, and 100 for brain. Inset, 8-fold enlargement of brain plot along the Y-axis. (DOCX) [file pone.0221886.s007.docx]

0

20

40

60

80

100

0

20

40

60

80

**µg protein**

**nanomole ATP hydrolyzed per min**

brain

muscle

liver

heart

2

4

0

20

10

brain

**S7 Fig. Steady-state rate of ATP hydrolysis sensitive to (IF1 + oligomycin) as a function of the protein amount.**

Conditions as in S4 Fig; four homogenates of different frozen-thawed tissues from heart (●), liver (○), muscle (■) and brain (□); specific activities expressed as nmol ATP hydrolyzed per min and per mg protein were 1390 for heart, 427 for liver, 312 for muscle, and 100 for brain. Inset, 8-fold enlargement of brain plot along the Y-axis.
