# Supplementary material for: Kinetic analysis of ATP hydrolysis by complex V in four murine tissues: Towards an assay suitable for clinical diagnosis
Source: PLoS One. 2019 Aug 28;14(8):e0221886. doi: 10.1371/journal.pone.0221886 (PMC6713359; doi:10.1371/journal.pone.0221886)
Supplement: S8 Fig — Conditions as in S4 Fig; homogenates of different frozen-thawed tissues from brain, liver, muscle and heart; each data point = average of two measurements; different symbols (■, □, ▲, Δ) = independent preparations (four for brain, two for liver and heart, three for muscle. We corrected MgATP concentrations for endogenous ATP as indicated in S3 Fig. For each preparation, we averaged two rate measurements performed at each MgATP concentration. Data were then fitted with the Michaelis-Menten equation. The rates at given MgATP concentrations were normalized to Vmax for each preparation and the normalized values from the different preparations were merged and fitted again with the Michaelis-Menten equation; continuous line = data fitting with the mean computed value of Km; dashed lines = data fitting with the mean value of Km plus or minus the standard deviation. Estimated Km values (μM): 148 ± 12 (brain); 220 ± 22 (liver); 217 ± 19 (muscle); 146 ± 11 μM (heart). (DOCX) [file pone.0221886.s008.docx]

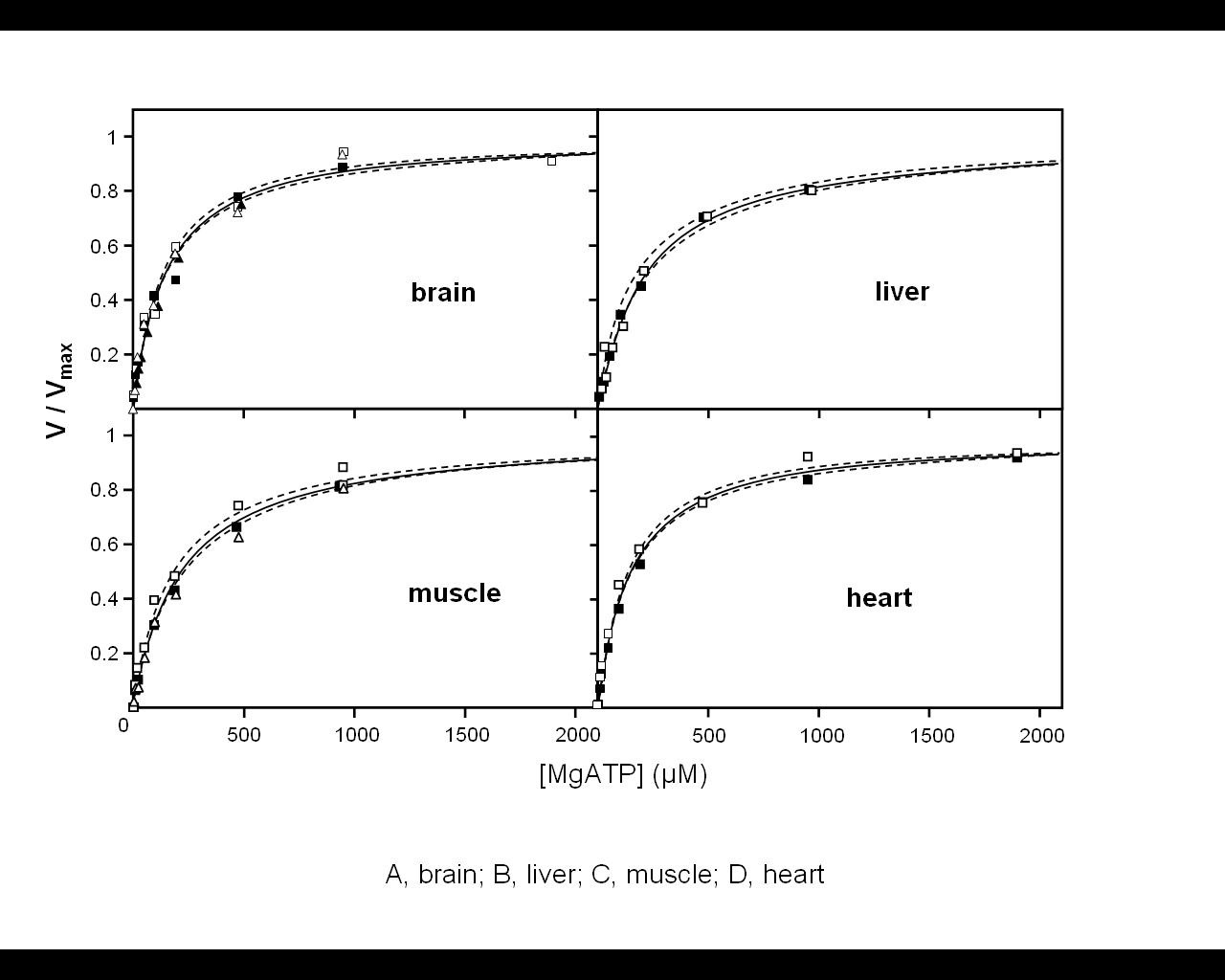


**S8 Fig. Normalized rate of ATP hydrolysis sensitive to (IF1 + oligomycin) as a function of MgATP concentration.**

Conditions as in S4 Fig; homogenates of different frozen-thawed tissues from brain, liver, muscle and heart; each data point = average of two measurements; different symbols (■, □, ▲, ∆) = independent preparations (four for brain, two for liver and heart, three for muscle. We corrected MgATP concentrations for endogenous ATP as indicated in S3 Fig. For each preparation, we averaged two rate measurements performed at each MgATP concentration. Data were then fitted with the Michaelis-Menten equation. The rates at given MgATP concentrations were normalized to V_max_ for each preparation and the normalized values from the different preparations were merged and fitted again with the Michaelis-Menten equation; continuous line = data fitting with the mean computed value of K_m_; dashed lines = data fitting with the mean value of K_m_ plus or minus the standard deviation. Estimated Km values (µM): 148 ± 12 (brain); 220 ± 22 (liver); 217 ± 19 (muscle); 146 ± 11 µM (heart).
