# Supplementary material for: Kinetic analysis of ATP hydrolysis by complex V in four murine tissues: Towards an assay suitable for clinical diagnosis
Source: PLoS One. 2019 Aug 28;14(8):e0221886. doi: 10.1371/journal.pone.0221886 (PMC6713359; doi:10.1371/journal.pone.0221886)
Supplement: S9 Fig — Conditions as in Fig 4 (main text); vertical arrows = additions (1 mM MgATP, 3 μM IF1, 6 μM oligomycin). First panel: protein content: 13 μg for brain, 9.5 μg for liver, 1.5 μg for heart, and 1.4 μg for muscle. Complex V activity expressed as nmol ATP hydrolyzed per min and per mg of protein was 925 for brain, 1122 for liver, 3104 for heart and 2691 for muscle. Second panel: comparison of kinetics obtained by adding MgATP about 2 min after the sample (upper trace) or about 30 min after (lower trace). That experience shows that the activation process proceeds in the absence as well as in the presence of MgATP. (DOCX) [file pone.0221886.s009.docx]

**S9 Fig. Time-course of ATP hydrolysis in presence of DDM by solubilized fractions from different organs.**

Conditions as in Fig 4 (main text); vertical arrows = additions (1 mM MgATP, 3 µM IF1, 6 µM oligomycin). First panel: protein content: 13 µg for brain, 9.5 µg for liver, 1.5 µg for heart, and 1.4 µg for muscle. Complex V activity expressed as nmol ATP hydrolyzed per min and per mg of protein was 925 for brain, 1122 for liver, 3104 for heart and 2691 for muscle. Second panel: comparison of kinetics obtained by adding MgATP about 2 min after the sample (upper trace) or about 30 min after (lower trace). That experience shows that the activation process proceeds in the absence as well as in the presence of MgATP.
