# Supplementary material for: Kinetic analysis of ATP hydrolysis by complex V in four murine tissues: Towards an assay suitable for clinical diagnosis
Source: PLoS One. 2019 Aug 28;14(8):e0221886. doi: 10.1371/journal.pone.0221886 (PMC6713359; doi:10.1371/journal.pone.0221886)
Supplement: S10 Fig — Conditions as in Fig 5 (main text). Upper panel = complex V immunoblot with samples of brain and muscle preparations at given complex V activities indicated on top of the lanes and expressed as nanomoles/minute. Lower panel = linear regression analysis of the signal intensities expressed as arbitrary units (A.U.) plotted against complex V activity; black symbols = samples from muscle, white symbols = signals from brain. The signals corresponding to 40 nanomoles ATP hydrolyzed per minute (between parentheses) diverged from linearity, being lower than expected. The deviation was significant for brain samples whose correlation coefficient was 0.66 when taking into account the four data points of the series and 0.96 when eliminating the data point corresponding to the highest activity. Linear regression analysis after withdrawal of the data points corresponding to the highest activity showed correlation coefficients close to 1 and a slope similar for brain and muscle preparations analyzed on the same gel. (DOCX) [file pone.0221886.s010.docx]

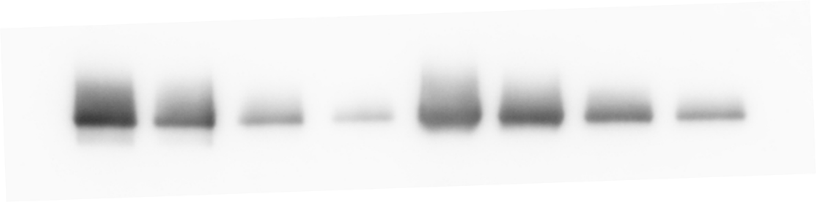


**5**

**10**

**20**

**40**

**Brain-1**

**Muscle-1**

**5**

**10**

**20**

**40**

**S10 Fig. Immunotitration of Complex V in solubilized fractions from brain and muscle homogenates**.

Conditions as in Fig 5 (main text). Upper panel = complex V immunoblot with samples of brain and muscle preparations at given complex V activities indicated on top of the lanes and expressed as nanomoles/minute. Lower panel = linear regression analysis of the signal intensities expressed as arbitrary units (A.U.) plotted against complex V activity; black symbols = samples from muscle, white symbols = signals from brain. The signals corresponding to 40 nanomoles ATP hydrolyzed per minute (between parentheses) diverged from linearity, being lower than expected. The deviation was significant for brain samples whose correlation coefficient was 0.66 when taking into account the four data points of the series and 0.96 when eliminating the data point corresponding to the highest activity.

Linear regression analysis after withdrawal of the data points corresponding to the highest activity showed correlation coefficients close to 1 and a slope similar for brain and muscle preparations analyzed on the same gel.
