# Supplementary material for: Kinetic analysis of ATP hydrolysis by complex V in four murine tissues: Towards an assay suitable for clinical diagnosis
Source: PLoS One. 2019 Aug 28;14(8):e0221886. doi: 10.1371/journal.pone.0221886 (PMC6713359; doi:10.1371/journal.pone.0221886)
Supplement: S11 Fig — Conditions as described under Materials and Methods; vertical arrows = sample (S) addition to the reaction medium; +10-UQ = presence of decyl-ubiquinone, a complex I substrate; no 10-UQ+rotenone = absence of the substrate and presence of rotenone, a complex I inhibitor; protein amount in the assay: 35 μg (brain), 47 μg (liver), 83 μg (muscle), 18 μg (heart). The dashed straight lines show the initial rates computed by fitting the first three minutes of the kinetics with a monoexponential curve. Complex I specific activity, expressed as nanomoles NADH oxidized per minute and per mg protein, was 169 for brain, 98 for liver, 168 for muscle, and 1164 for heart. NADH oxidation, unrelated to complex I, was 12% of the total activity for brain, 13% for liver, 1% for muscle, and 2% for heart. (DOCX) [file pone.0221886.s011.docx]

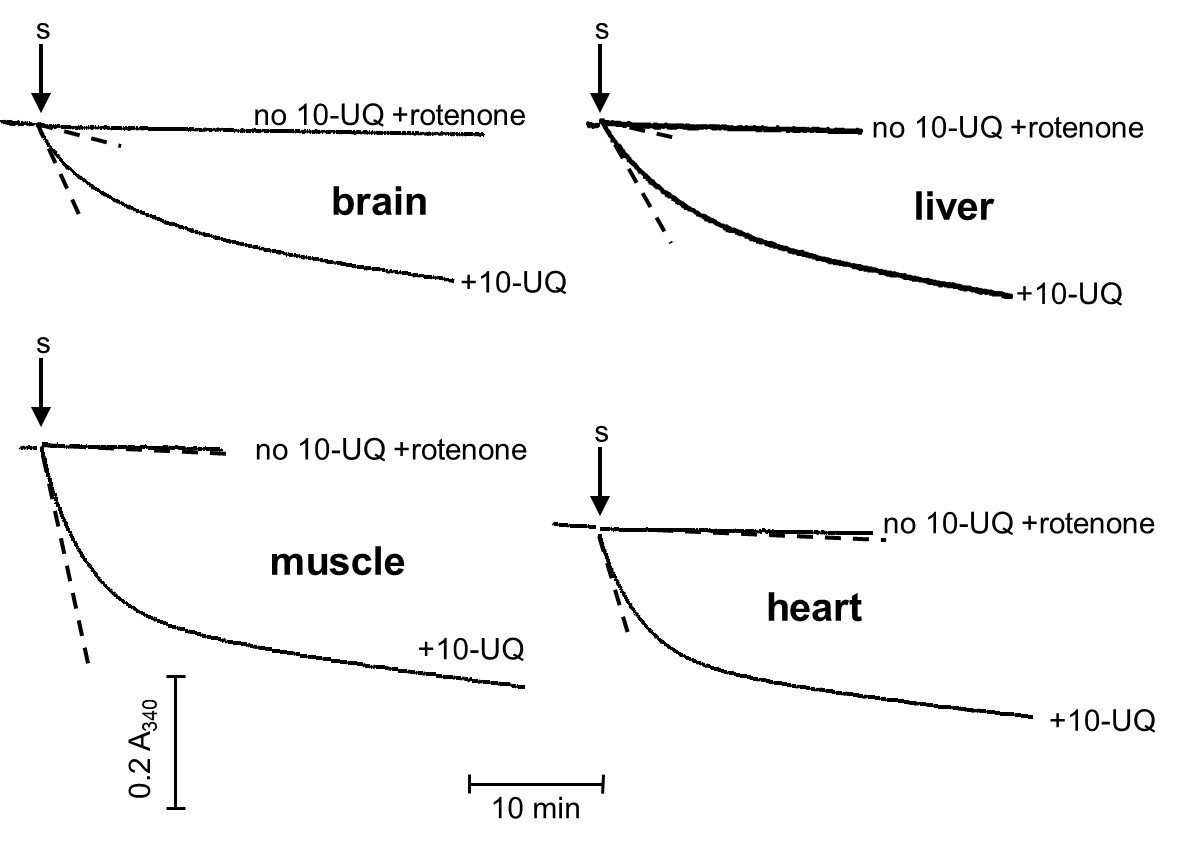


**S11 Fig. Time-course of complex I-dependent NADH oxidation by homogenates from different organs.**

Conditions as described under Materials and Methods; vertical arrows = sample (S) addition to the reaction medium; +10-UQ = presence of decyl-ubiquinone, a complex I substrate; no 10-UQ+rotenone = absence of the substrate and presence of rotenone, a complex I inhibitor; protein amount in the assay: 35 µg (brain), 47 µg (liver), 83 µg (muscle), 18 µg (heart). The dashed straight lines show the initial rates computed by fitting the first three minutes of the kinetics with a monoexponential curve. Complex I specific activity, expressed as nanomoles NADH oxidized per minute and per mg protein, was 169 for brain, 98 for liver, 168 for muscle, and 1164 for heart. NADH oxidation, unrelated to complex I, was 12% of the total activity for brain, 13% for liver, 1% for muscle, and 2% for heart.
