# Supplementary material for: Kinetic analysis of ATP hydrolysis by complex V in four murine tissues: Towards an assay suitable for clinical diagnosis
Source: PLoS One. 2019 Aug 28;14(8):e0221886. doi: 10.1371/journal.pone.0221886 (PMC6713359; doi:10.1371/journal.pone.0221886)
Supplement: S12 Fig — Conditions as described under Materials and Methods; vertical arrows = addition to the reaction medium of sample (S) or malonate, an inhibitor of complex II; protein amount in the assay: 108 μg (brain), 47 μg (liver), 83 μg (muscle), 9 μg (heart). Complex II specific activity, expressed as nanomoles NADH oxidized per minute and per mg protein, was 56 for brain, 137 for liver, 120 for muscle, and 847 for heart (DOCX) [file pone.0221886.s012.docx]

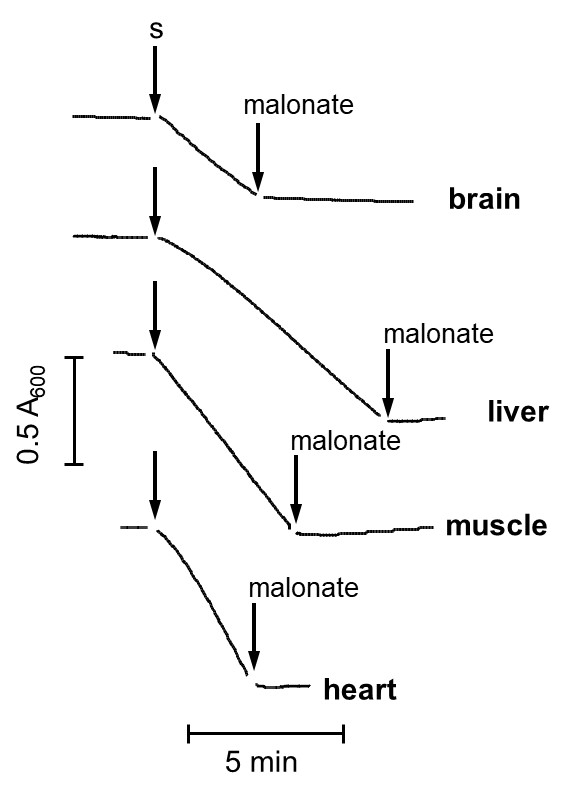


**S12 Fig. Time-course of complex II-dependent DCPIP reduction by homogenates from different organs.**

Conditions as described under Materials and Methods; vertical arrows = addition to the reaction medium of sample (S) or malonate, an inhibitor of complex II; protein amount in the assay: 108 µg (brain), 47 µg (liver), 83 µg (muscle), 9 µg (heart). Complex II specific activity, expressed as nanomoles NADH oxidized per minute and per mg protein, was 56 for brain, 137 for liver, 120 for muscle, and 847 for heart.
