# Supplementary material for: Kinetic analysis of ATP hydrolysis by complex V in four murine tissues: Towards an assay suitable for clinical diagnosis
Source: PLoS One. 2019 Aug 28;14(8):e0221886. doi: 10.1371/journal.pone.0221886 (PMC6713359; doi:10.1371/journal.pone.0221886)
Supplement: S13 Fig — Conditions as described under Materials and Methods; vertical arrows = addition to the reaction medium of 1 mM MgATP, 3 μM IF1 or 6 μM oligomycin. ATP hydrolysis activity, expressed as nanomoles ATP hydrolyzed per minute and per mg protein, was 385 for a fragment of gluteus maximus (upper trace, 16 μg protein in the assay) and 354 for a fragment of gluteus minimus (lower trace, 34 μg protein in the assay). In both cases, IF1 + oligomycin inhibited 88% of the activity. (DOCX) [file pone.0221886.s013.docx]

**S13 Fig. Time-course of complex V assay in human muscle.**

Conditions as described under Materials and Methods; vertical arrows = addition to the reaction medium of 1 mM MgATP, 3 µM IF1 or 6 µM oligomycin. ATP hydrolysis activity, expressed as nanomoles ATP hydrolyzed per minute and per mg protein, was 385 for a fragment of gluteus maximus (upper trace, 16 µg protein in the assay) and 354 for a fragment of gluteus minimus (lower trace, 34 µg protein in the assay). In both cases, IF1 + oligomycin inhibited 88% of the activity.
